# Hormonal Modulation in Chronic Coronary Syndrome: From Neurohormonal Activation to Endocrine Targets

**DOI:** 10.3390/biomedicines14091954

**Published:** 2026-08-30

**Authors:** Alina Diduța Brie, Roxana Popescu, Cristian Mornoș, Daniel Miron Brie

**Affiliations:** 1 Department of Cell and Molecular Biology, “Victor Babeș” University of Medicine and Pharmacy, Tudor Vladimirescu Street, No. 14, 300174 Timișoara, Romania; alina.brie@umft.ro (A.D.B.); popescu.roxana@umft.ro (R.P.); 2 ANAPATMOL Research Center, “Victor Babeș” University of Medicine and Pharmacy, Tudor Vladimirescu Street, No. 14, 300174 Timișoara, Romania; 3 “Louis Țurcanu” Emergency Children Hospital, Doctor Iosif Nemoianu Street, No. 2, 300011 Timișoara, Romania; 4 Cardiovascular Disease Institute Timișoara, Gheorghe Adam Street, No. 13A, 300310 Timișoara, Romania; mornos.cristian@umft.ro; 5 Research Center of the Institute of Cardiovascular Diseases, Gheorghe Adam Street, No. 13A, 300310 Timișoara, Romania; 6 Department of Cardiology, “Victor Babeș” University of Medicine and Pharmacy Timișoara, Eftimie Murgu Square, No. 2, 300041 Timișoara, Romania

**Keywords:** chronic coronary syndrome, neurohormonal activation, renin–angiotensin–aldosterone system, beta-blockers, natriuretic peptides, sex hormones, thyroid hormones, GLP-1 receptor agonists, SGLT2 inhibitors

## Abstract

**Background:** Chronic coronary syndrome (CCS) has evolved from a stenosis-centered concept to a dynamic clinical entity in which obstructive and non-obstructive coronary disease, microvascular dysfunction, and vasomotor abnormalities coexist with complex neurohormonal and endocrine activation. However, CCS has rarely been examined through an integrated endocrine lens, and no unified hormonal-phenotype framework currently links neurohormonal, sex-hormone, thyroid, and cardiometabolic pathways across CCS presentations. **Objective:** To synthesize contemporary evidence on how neurohormonal and endocrine pathways shape CCS phenotypes and to outline potential endocrine targets for phenotype-guided therapy. **Methods for Synthesis of Data:** We performed a structured literature appraisal in PubMed, Embase, the Cochrane Library, and ScienceDirect from inception to May 2026, using CCS/stable coronary artery disease and hormone-related search terms, and included comparative clinical studies in adults that evaluated neurohormonal or endocrine interventions with clinically relevant cardiovascular outcomes. **Results:** From 851 records, 34 studies were retained for qualitative synthesis, encompassing randomized trials, observational cohort studies, systematic reviews, and consensus documents. These data support a central role for renin–angiotensin–aldosterone and sympathetic activation, counterregulatory natriuretic peptides, endothelin-1, sex and thyroid hormones, and adipose-derived mediators in modulating CCS phenotypes, symptoms, and residual risk. Hormone-targeting therapies—including RAAS blockade, beta-blockers, mineralocorticoid receptor antagonists, sex- and thyroid-hormone modulation, and cardiometabolic agents such as GLP-1 receptor agonists and SGLT2 inhibitors—show variable yet often significant impact on outcomes across specific CCS subsets. **Conclusions:** CCS is best conceptualized as a state of coordinated neurohormonal and endocrine disturbance rather than a purely anatomical disease. A hormonal phenotype framework may support more biologically rational, individualized management and define a research agenda for phenotype-stratified trials in CCS. This review proposes a hormonal-phenotype framework that is conceptually novel compared with anatomy- or comorbidity-based CCS classifications and is intended to be hypothesis-generating rather than prescriptive, by highlighting specific knowledge gaps and potential endocrine targets for future phenotype-guided trials.

## 1. Introduction

Chronic coronary syndrome (CCS) is currently understood as the clinical expression of coronary artery disease during stable periods—including phases that precede or follow an acute coronary syndrome—rather than as a static condition defined only by fixed epicardial stenosis [1,2]. This contemporary view emphasizes that CCS is a dynamic disorder in which symptoms, ischemic burden, plaque behavior, vascular tone, and event risk may fluctuate over time, even without an acute presentation. The 2024 ESC framework explicitly extends the concept beyond obstructive epicardial disease to include non-obstructive coronary atherosclerosis, coronary microvascular dysfunction (CMD), and vasomotor abnormalities, all of which may sustain recurrent ischemia and persistent symptoms [1].

From an epidemiological perspective, CCS remains a major contributor to global cardiovascular morbidity and mortality [3]. Although contemporary management combines lifestyle intervention, lipid-lowering, antithrombotic therapy, antianginal treatment, and selective revascularization, many patients continue to experience major adverse cardiovascular events (MACE), persistent angina, heart failure (HF) progression, and impaired quality of life because of a substantial residual cardiovascular risk [3,4]. This residual risk is increasingly recognized as heterogeneous and multifactorial, with thrombotic, inflammatory, metabolic, and procedural mechanisms that interact variably in individual patients despite apparently optimal guideline-directed therapy [3,5].

Against this background, CCS should not be viewed solely as a mechanical disease of luminal narrowing. Coronary pathology evolves within a broader biological milieu characterized by chronic neurohormonal and endocrine activation that influences endothelial function, vascular inflammation, smooth-muscle tone, coronary microcirculatory regulation, myocardial energetics, and ventricular remodeling [6,7,8]. Even subtle disturbances in hormonal systems may contribute directly to atherogenesis, plaque instability, thrombosis, and maladaptive cardiovascular signalling, while also acting indirectly through blood-pressure dysregulation, insulin resistance, adipose dysfunction, and altered lipid metabolism [7,9]. This review addresses a key gap: the lack of a unified, hormone-centric model for CCS. Existing guidelines and trials primarily focus on anatomy, ischemia burden, and comorbidities, while hormonal axes are treated as parallel conditions rather than core determinants of CCS phenotypes and residual risk. We therefore propose an integrative hormonal-phenotype framework that links neurohormonal, sex-hormone, thyroid, and cardiometabolic pathways to clinically relevant CCS subsets. The aim is to provide a biologically coherent scaffold for hypothesis generation, risk stratification, and future phenotype-guided trials, complementing rather than replacing current ESC CCS scenarios.

Classical neurohormonal axes—the renin–angiotensin–aldosterone system (RAAS) and the sympathetic nervous system—coordinate short-term hemodynamic adaptation, yet chronic activation promotes vasoconstriction, oxidative stress, inflammation, fibrosis, and adverse remodeling across the vascular wall and myocardium [6,10]. Counter-regulatory pathways, particularly the natriuretic peptides (NPs), exert vasodilatory, natriuretic, and RAAS-antagonizing effects, but their protective capacity may become insufficient in chronic cardiometabolic disease states [11,12]. In parallel, endothelin signaling, adipokine imbalance, and broader metabolic-endocrine networks further modulate vascular reactivity and tissue injury, strengthening the notion that coronary disease is embedded in an endocrine and paracrine continuum rather than limited to plaque anatomy alone [7,9].

Neurohormonal activation is firmly established as a central mechanism in HF, in which sympathetic, RAAS, vasopressin, and natriuretic peptide pathways are closely linked to disease progression, biomarker development, and treatment response [13]. By contrast, its role in CCS has been discussed less systematically, often under the generic umbrella of coronary artery disease (CAD), without sufficiently distinguishing among clinically relevant CCS phenotypes [3,7]. Phenotypes such as microvascular angina, diffuse non-obstructive CAD, and post-PCI CCS likely differ not only in anatomic substrate but also in the balance of endothelial dysfunction, microvascular constriction, inflammation, metabolic stress, and neurohormonal signaling [14,15].

This narrative review addresses CCS as a dynamic coronary disorder shaped by intertwined neurohormonal and endocrine mechanisms. It moves beyond a stenosis-centered narrative to examine how hormonal pathways influence vascular biology, coronary microcirculation, myocardial metabolism, symptom persistence, and residual risk across distinct CCS phenotypes [1,6,7]. Recent heart–brain axis reviews highlight how subclinical cardiac and aortic alterations, autonomic imbalance, and humoral signaling contribute to cognitive vulnerability and neurological outcomes, underscoring CCS as part of a systemic cardio-neuro-endocrine continuum rather than an isolated vascular disease [16,17,18].

By integrating classical neurohormonal systems with endocrine targets such as sex, thyroid, and metabolic hormones—and by incorporating recent randomized and observational evidence on hormone-modulating therapies—the review aims to provide a coherent pathophysiological framework for understanding heterogeneity in CCS, and to identify translational opportunities for risk stratification and future therapy [7,9,19].

## 2. Methods for Synthesis of Data

This work is best characterized as a narrative review with a structured literature search, rather than a fully systematic review. The review employed predefined eligibility criteria, multiple databases, independent screening, and a PRISMA-style flow diagram to enhance transparency, but it was not prospectively registered (e.g., in PROSPERO) and did not adhere to all PRISMA requirements. No quantitative meta-analysis was undertaken because of substantial heterogeneity in study design, endpoint definitions, and follow-up schedules across the included literature.

**Figure 1:**
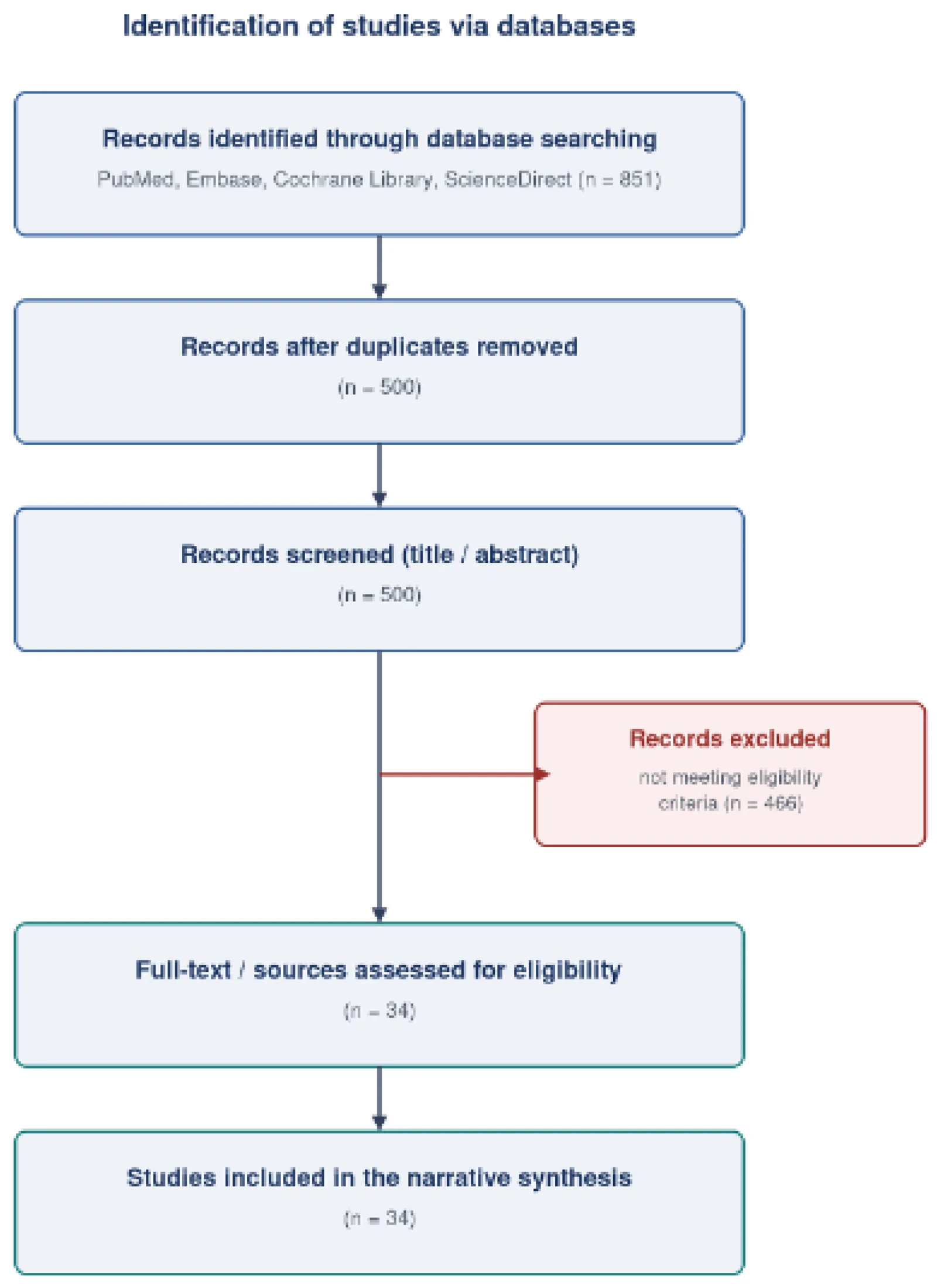
PRISMA-style flow diagram of study identification, screening, and inclusion. From 851 records identified across PubMed, Embase, the Cochrane Library, and ScienceDirect, 500 unique studies were screened and 34 were retained for the narrative synthesis.

This article is a narrative review structured around a pathophysiology-to-therapy framework, with particular emphasis on biologically active hormonal and cardiometabolic interventions in CCS. To anchor the narrative in contemporary evidence, we performed a structured literature appraisal across PubMed, Embase, the Cochrane Library, and ScienceDirect from database inception through May 2026; the identification-to-inclusion process is summarized in a PRISMA-style flow diagram (Figure 1) [19,20]. Supplementary Table S1 will provide the full search strategy: databases searched, exact search dates, date limits (from database inception to 31 May 2026), English-language restriction, and complete PubMed MeSH/free-text and Embase Emtree/free-text search strings.

The literature search was restricted to English-language articles published in peer-reviewed journals. When multiple reports originated from the same trial or registry, we preferentially included the most informative and/or latest publication, while using earlier reports only if they provided unique mechanistic or subgroup data. To preserve methodological consistency and avoid selective outcome reporting, we did not systematically include grey literature such as conference abstracts, theses, or unpublished trial-registry entries. Grey literature was therefore considered only when essential for contextualizing ongoing or emerging therapies, and not as part of the core evidentiary dataset. Search terms combined CCS- and stable-coronary-disease descriptors with terms related to neurohormonal and endocrine pathways, including RAAS inhibitors, beta-blockers, mineralocorticoid receptor antagonists, thyroid hormones, sex hormones, GLP-1 receptor agonists, and SGLT2 inhibitors, together with cardiovascular outcomes. In this narrative review, studies were considered eligible for inclusion if they enrolled adult patients with chronic coronary syndrome or stable coronary artery disease, evaluated at least one hormonal or neurohormonal intervention (such as renin–angiotensin–aldosterone system inhibitors, beta-blockers, mineralocorticoid receptor antagonists, thyroid or sex-hormone–targeted therapies, or cardiometabolic biologics including GLP-1 receptor agonists and SGLT2 inhibitors), and reported clinically relevant outcomes such as mortality, major adverse cardiovascular events, myocardial infarction, heart-failure hospitalization, cardiac function parameters, or validated symptom and quality-of-life measures. Comparative randomized controlled trials, cohort and case–control studies, registries, systematic reviews, meta-analyses, and evidence-based consensus documents were retained, whereas reports restricted to acute coronary syndromes without longitudinal follow-up in the chronic phase, non-comparative case series or case reports, preclinical or in vitro experiments, and studies in which the primary intervention did not directly modulate a hormonal or neurohormonal axis (for example, isolated revascularization procedures or antiplatelet/statin trials without endocrine analysis) were excluded, as were articles limited to surrogate biomarkers without clinical or functional endpoints. Eligibility criteria prioritized studies focused on CCS or stable coronary artery disease, comparative clinical designs, and clinically relevant outcomes, while excluding studies restricted to acute coronary syndromes, non-comparative reports, preclinical models, and non-hormonal primary interventions. From 851 identified records, 500 unique studies were screened, and 34 were retained after two-stage selection, comprising randomized trials, observational cohorts, systematic reviews, retrospective studies, and consensus documents that informed the present narrative synthesis. The 34 studies retained through this structured appraisal formed the evidentiary core for the therapy-focused sections; additional background, mechanistic, and guideline references are cited throughout the narrative, which accounts for the larger total reference count. Although the article is narrative rather than systematic and does not provide pooled effect estimates or formal risk-of-bias grading, this structured evidence base was used to strengthen the sections on hormone-targeting therapies, especially the growing role of cardiometabolic biologics within phenotype-guided CCS management. Supplementary Table S2 lists the 34 core therapy-focused sources included in the structured appraisal, with study design, population, coronary context, intervention/exposure, outcome domain, and relevance to the review.

Study selection and evidence appraisal. Two authors independently screened titles and abstracts, followed by full-text assessment of potentially eligible studies, with disagreements resolved by discussion and, when needed, input from a third reviewer. We prioritized comparative clinical designs with clinically relevant endpoints and gave greater weight in the narrative synthesis to randomized controlled trials and large prospective cohorts, while using smaller or observational studies primarily for mechanistic context. Because of the heterogeneity of study designs, populations, hormonal interventions, and outcome definitions, we did not perform a formal risk-of-bias grading or meta-analysis; instead, we qualitatively appraised internal validity, consistency, and biological plausibility when interpreting treatment effects and constructing the hormonal-phenotype framework.

This review was conceived as a narrative synthesis, informed by a prespecified, structured literature search across major biomedical databases rather than as a full systematic review. We did not perform a formal risk-of-bias assessment or meta-analysis because the included evidence spans highly heterogeneous study designs, patient populations (CCS, stable CAD, HF, cardiometabolic cohorts), and endpoints, which preclude meaningful pooling of effect sizes. Instead, we prioritized mechanistic interpretation and pathophysiology-to-therapy linkage, using trial and observational data qualitatively to support a conceptual “hormonal-phenotype” framework. This approach aligns with the article’s primary aim: to generate biologically coherent hypotheses and clinical perspectives rather than to provide quantitative-effect estimates. This narrative approach allows integration of heterogeneous mechanistic and clinical data into a coherent pathophysiology-to-therapy framework but carries important methodological limitations. Without formal risk-of-bias grading, effect-size pooling, or certainty-of-evidence assessment, our conclusions about specific hormonal interventions must be interpreted as qualitative and hypothesis-generating, and the relative strength of evidence across axes (for example RAAS inhibitors versus sex-hormone therapies) should be viewed as approximate rather than quantitatively ranked.

This approach aligns with current recommendations for transparent narrative reviews and is not intended to meet the full methodological standards of a systematic review or meta-analysis.

## 3. Hormonal Responses in CCS

CCS is accompanied by a coordinated hormonal response to chronic ischemic and cardiometabolic stress, integrating classical neurohormonal systems, sex and thyroid hormones, and adipose-derived mediators. Key components include the renin–angiotensin–aldosterone system, the sympathetic–adrenal axis, natriuretic peptides, endothelin-1, sex steroids, thyroid hormones, and cardiometabolic hormones such as insulin, incretins, and adipokines, which together shape endothelial function, coronary microcirculation, myocardial energetics, and ventricular remodeling. Although inflammation is a central component of CCS pathophysiology and residual risk, detailed coverage of specific inflammatory pathways and anti-inflammatory therapies (e.g., IL-1/IL-6 targeting) falls outside the primary scope of this hormone-focused review; instead, we discuss inflammatory mechanisms insofar as they interact with the neurohormonal and endocrine axes outlined here. Inflammation should be considered a linked, cross-cutting domain rather than a discrete endocrine CCS phenotype. Low-grade systemic and vascular inflammation may interact bidirectionally with neurohormonal activation, endothelial dysfunction, insulin resistance, adipose-tissue dysregulation, and coronary microvascular impairment, thereby contributing to residual risk and symptom burden across obstructive CAD, ANOCA/INOCA, and cardiometabolic CCS. Its clinical relevance is therefore best framed as a mechanistic and prognostic modifier that may inform risk stratification and preventive intensification, while not replacing phenotype-specific diagnostic evaluation or guideline-directed treatment.

### 3.1. Renin–Angiotensin–Aldosterone System

The renin–angiotensin–aldosterone system is one of the best-characterized neurohormonal networks in cardiovascular disease and provides a biologically plausible bridge between chronic coronary atherosclerosis and HF progression [6,13]. In physiological conditions, RAAS maintains arterial pressure, sodium balance, and tissue perfusion; persistent activation, however, shifts this adaptive system toward vascular injury and maladaptive remodeling [6,13]. Angiotensin II promotes vasoconstriction, endothelial dysfunction, oxidative stress, inflammatory gene activation, smooth-muscle proliferation, and platelet–thrombotic activation, while aldosterone amplifies sodium retention, vascular inflammation, collagen deposition, and myocardial fibrosis [6,13,21]. Within the coronary circulation, these effects reduce nitric-oxide bioavailability, increase reactive oxygen species, and accelerate structural changes in both macro- and microvascular compartments, contributing to plaque progression and impaired vasomotor reserve [6,22,23].

Clinical data support the relevance of RAAS in stable coronary disease. ACE inhibition reduced major adverse cardiovascular events versus placebo in the HOPE trial (*n* = 9297), reducing the composite of cardiovascular death, MI, and stroke by 22% in high-risk vascular patients without overt HF [24], and perindopril decreased the composite of cardiovascular death, myocardial infarction (MI), and cardiac arrest by 20% in stable CAD in EUROPA (*n* = 12,218) [25]. In contemporary populations on intensive background therapy, the absolute benefit of RAAS inhibition is smaller [26], but its place as foundational therapy is reinforced by data showing cardiovascular benefit even in patients with advanced chronic kidney disease (CKD), where concerns about acute kidney injury (AKI) and hyperkalemia are most acute [26,27]. A large multicenter retrospective study reported that ACEI/ARB use was associated with lower total and cardiovascular mortality in CAD patients with advanced CKD, supporting continued—though carefully monitored—RAAS blockade in this high-risk subgroup [27].

The persistence of RAAS signaling despite ACE-inhibitor or angiotensin receptor blocker (ARB) therapy is mechanistically important: alternative enzymatic pathways (e.g., chymase and cathepsins) can continue to generate angiotensin II locally, and aldosterone concentrations may “escape” during long-term blockade [28,29]. Residual RAAS activity may therefore sustain endothelial dysfunction, microvascular constriction, oxidative stress, and low-grade vascular inflammation in apparently optimally treated patients [6,28]. This concept is particularly relevant in CCS, where residual risk persists despite contemporary lipid-lowering, antithrombotic therapy, and blood-pressure control. Mechanistically, several CCS phenotypes are linked to RAAS activation. In obstructive and diffuse atherosclerotic CCS, angiotensin II enhances vascular smooth-muscle proliferation, extracellular-matrix remodeling, and plaque inflammation [6,22]. In microvascular angina and INOCA-related CCS, RAAS may contribute to impaired coronary flow reserve through endothelial dysfunction, reduced nitric-oxide signaling, heightened vasoconstrictor tone, and structural rarefaction of resistance vessels [14,15,23]. In post-PCI CCS, persistent RAAS activation intersects with vascular healing, restenosis biology, and recurrent ischemic symptoms by maintaining a pro-inflammatory and pro-fibrotic milieu beyond the treated lesion [6,25,30]. Most RAAS-related clinical data referenced in this section originate from mixed CAD, high-risk vascular, or HF cohorts, with only a subset specifically designed for CCS or stable coronary populations. Therefore, extrapolation to CCS phenotypes without HF must be interpreted with caution. In clinical practice, RAAS activation in CCS is inferred indirectly from comorbidities (hypertension, diabetes, CKD), biomarker patterns (e.g., persistent natriuretic peptide elevation despite preserved EF), and imaging features of diffuse atherosclerosis and remodeling rather than from routine measurement of RAAS components. Within the proposed framework, these features may help identify patients with a “RAAS-associated biological profile” CCS phenotype in whom optimized RAAS blockade and careful monitoring might be prioritized, although this concept requires validation in CCS-specific trials.

### 3.2. Sympathetic–Adrenal Axis and Catecholamines

The sympathetic-adrenal system is another central component of the neurohormonal response in CCS [10,13]. Chronic adrenergic drive shapes coronary disease by increasing heart rate, blood-pressure variability, myocardial oxygen demand, vasoconstrictor tone, platelet activation, and electrical instability [10,13]. In patients with coronary atherosclerosis or endothelial dysfunction, these effects are particularly relevant because the ischemic threshold may be lowered not only by fixed stenosis but also by dynamic changes in vascular tone and microvascular resistance [14,22,23].

Increased sympathetic tone augments α-adrenergic vasoconstriction, especially during exercise, cold exposure, or mental stress, and this may be exaggerated when endothelial nitric-oxide signaling is impaired [14]. Sympathetic activation also accelerates heart rate and shortens diastole, reducing coronary perfusion time while increasing myocardial work [14,22]. Resting tachycardia and exaggerated heart-rate responses are markers of autonomic imbalance and reflect a more activated adrenergic phenotype, while blood-pressure lability can further increase shear stress and endothelial injury [10,22]. Importantly, autonomic-function studies in CCS with preserved ejection fraction have shown that hyper-adrenergic tone persists in a sizeable proportion of patients despite established beta-blocker therapy, suggesting that conventional dosing may be insufficient to fully neutralize sympathetic drive [31].

The role of sympathetic activation is especially relevant in functional coronary disorders. In CMD, an augmented α-adrenergic constrictor response during sympathetic stimulation contributes to ischemia and reduced flow reserve [14,23]. This observation is pertinent to CCS phenotypes such as microvascular angina or stress-induced recurrent angina after apparently successful revascularization, where the burden of symptoms is poorly explained by angiographic anatomy alone [14,23].

### 3.3. Natriuretic Peptides and Counter-Regulatory Hormones

Natriuretic peptides represent the major endogenous counter-regulatory system opposing RAAS and sympathetic activation [11,12]. Atrial natriuretic peptide and B-type natriuretic peptide (BNP) promote natriuresis, vasodilation, inhibition of renin and aldosterone release, and antifibrotic signaling, thereby buffering the hemodynamic and structural consequences of chronic neurohormonal stress [11,12]. In HF, BNP and N-terminal proBNP (NT-proBNP) are established markers of wall stress and prognosis [13]; their relevance, however, extends beyond overt pump failure into the chronic coronary domain.

In CCS, elevations of BNP or NT-proBNP may capture subclinical myocardial stress even when frank HF is absent. Chronic ischemia, repeated transient supply–demand mismatch, diastolic dysfunction, diffuse fibrosis, microvascular dysfunction, and subtle left-ventricular remodeling can all increase myocardial wall tension sufficiently to activate natriuretic-peptide signaling [32,33]. Available evidence supports a prognostic role across ischemic syndromes: in the PEACE trial substudy, BNP and NT-proBNP added independent prognostic value beyond conventional risk factors in patients with stable CAD [32], and the Heart and Soul Study showed that NT-proBNP predicted cardiovascular events and mortality in stable coronary patients [33]. Natriuretic peptides are therefore conceptually attractive as biomarkers of the broader ischemic-neurohormonal state rather than as exclusive markers of symptomatic HF [12,32].

### 3.4. Endothelin-1 and Other Vasoactive Peptides

Endothelin-1 (ET-1) is one of the most potent endogenous vasoconstrictors and occupies a key position at the interface between endothelial dysfunction, inflammation, vascular remodeling, and coronary microvascular disease [22,34]. Beyond vasoconstriction, ET-1 exerts pro-inflammatory, mitogenic, oxidative, and profibrotic effects that amplify vascular injury within both epicardial and resistance vessels [22,34]. ET-1 therefore behaves not as an accessory pathway but as a plausible hormonal effector of persistent ischemia in CCS phenotypes marked by endothelial dysfunction and abnormal vasomotion [23,35].

Human data support the clinical relevance of this pathway. Genetic dysregulation of the endothelin pathway has been linked to CMD, with higher ET-1 levels and a greater likelihood of microvascular disease in patients with ischemic symptoms but no obstructive CAD [34]. The PRIZE trial of zibotentan, a selective ETA antagonist, has been designed specifically to test whether endothelin blockade improves outcomes in microvascular angina [35]. While endothelin receptor antagonism is not yet established therapy for CCS, it represents one of the most promising examples of endocrine–vascular targeting in patients whose disease is driven by microvascular dysfunction and vasomotor dysregulation [34,35]. While these data support a mechanistic role for ET-1 in microvascular angina and CCS-related vasomotor dysfunction, the clinical utility of routine ET-1 measurement and endothelin receptor blockade in CCS remains investigational. Ongoing trials such as PRIZE will clarify whether endothelin antagonism improves symptoms or outcomes in selected functional CCS phenotypes, but at present this strategy cannot be recommended for routine CCS management.

### 3.5. Integrative Perspective

Taken together, RAAS activation, sympathetic–adrenal signaling, natriuretic-peptide release, and endothelin excess support the idea that CCS may carry a measurable neurohormonal signature analogous in concept—though not identical—to the neurohormonal staging used in HF [12,13]. In CCS, such a signature would relate less to congestion and low output and more to ischemia burden, endothelial dysfunction, plaque activity, coronary microvascular impairment, and the transition from compensated chronic ischemia toward mixed ischemic-remodeling phenotypes [6,14,22]. Combined profiling of RAAS-related markers, natriuretic peptides, autonomic indices, and endothelin-related signals could theoretically identify patients with a more active biological phenotype than traditional anatomical scores suggest, especially in diffuse non-obstructive CAD, microvascular angina, diabetes-associated CCS, and post-PCI angina [12,14,23,34].

The hormonal-phenotype framework proposed in this review is intended to complement, rather than replace, the 2024 ESC CCS guidelines, which organize care around clinical scenarios (e.g., angina with suspected CCS, CCS with prior MI/PCI/CABG) and major comorbidity modules. By mapping neurohormonal, sex-hormone, thyroid, and cardiometabolic axes onto these scenarios, our approach highlights biologically distinct CCS subsets, including microvascular and vasomotor angina, post-ACS CCS with adverse remodeling, and cardiometabolic CCS with obesity, diabetes, or HFpEF predominance. Within each ESC scenario, hormonal profiling is proposed as an additional layer that may refine risk stratification and therapeutic prioritization (for example, emphasizing GLP-1RAs/SGLT2is in cardiometabolic CCS, careful RAAS/ET-1 targeting in microvascular or post-PCI CCS, or systematic assessment of thyroid and sex-hormone status in selected subgroups). These concepts extend beyond current guideline recommendations by suggesting a future, phenotype-guided paradigm in which endocrine and neurohormonal signatures help to individualize treatment choices within an otherwise guideline-directed CCS framework.

## 4. Sex Hormones and CCS

### 4.1. Epidemiological Observations

CCS displays clear sex differences that implicate endogenous sex hormones in modulating long-term coronary risk. Premenopausal women have a substantially lower prevalence of obstructive CAD and clinical coronary events than age-matched men, with the female advantage attenuating and eventually disappearing after the menopausal transition [36,37]. Observational and mechanistic data support the concept that endogenous estrogens exert predominantly anti-atherogenic and vasculoprotective actions, including favorable effects on vascular tone, lipids, and inflammatory pathways [36]. Cross-sectional studies of men with established CAD often report lower total and free testosterone and relatively higher estradiol concentrations than in healthy controls, but prospective associations between baseline sex-hormone levels and incident events have been inconsistent [38,39]. Sex-hormone status is therefore best interpreted within a broader cardiometabolic context rather than as an isolated linear risk factor [38,40].

Beyond estrogen and testosterone, follicle-stimulating hormone (FSH) has emerged as an under-recognized modulator of cardiovascular risk in postmenopausal women: elevated FSH levels are associated with disrupted lipid metabolism, exacerbated vascular inflammation, and progression of coronary atherosclerosis, raising the possibility that FSH, rather than estrogen deficiency alone, mediates part of the menopause-associated increase in CCS risk [41]. From a CCS perspective, the loss of estrogen-related protection in women coincides with rising rates of chronic coronary events and a shift towards more diffuse, microvascular, and non-obstructive ischemic phenotypes [15,42]. In men, low testosterone and disturbed sex hormone-binding globulin (SHBG) are linked to frailty, reduced exercise capacity, and impaired quality of life, which may modulate symptom burden and functional status in CCS independently of anatomy [39,43].

### 4.2. Mechanistic Pathways

Sex steroids influence the full continuum from cardiometabolic risk factors to vascular biology and myocardial remodeling. Estrogens improve lipid profiles by lowering LDL-cholesterol and raising HDL-cholesterol; androgens have more variable effects, depending on dose, formulation, and metabolic context [36,43]. At the vascular level, estrogens enhance nitric-oxide bioavailability, improve endothelium-dependent vasodilation, and attenuate oxidative stress and pro-inflammatory signaling through genomic and non-genomic pathways in endothelial and smooth-muscle cells [22,36]. Androgens act in a context-dependent manner: physiological testosterone supports lean mass, insulin sensitivity, and a more favorable visceral fat distribution in men, whereas both deficiency and supraphysiologic exposure may produce dyslipidemia, visceral adiposity, and endothelial dysfunction [39,43].

Sex steroids also interact with local cardiac and vascular neurohormonal systems. Preclinical evidence indicates that estrogens inhibit cardiac mast-cell activation and chymase release, thereby limiting local angiotensin-II generation, matrix degradation, and adverse remodelling [44]. Experimental and translational work suggests that estrogens enhance coronary microvascular function, reduce vasospasm, and improve endothelial responses—actions that are central to microvascular angina and vasomotor CCS phenotypes [14,42]. From a phenotyping perspective, sex-hormone status may help explain diffuse non-obstructive and microvascular CCS presentations in postmenopausal women and frail, hypogonadal men, and could inform individualized risk assessment when combined with traditional factors and imaging. However, robust data on routine sex-hormone profiling to guide CCS management are limited, and in the present framework sex hormones are primarily considered as modifiers of phenotype and prognosis rather than established therapeutic targets. Beyond estrogen withdrawal, the menopausal transition is accompanied by a marked rise in follicle-stimulating hormone (FSH), which may represent an additional and incompletely characterized component of vascular risk. Experimental and observational data suggest that FSH may influence lipid handling, adipose-tissue biology, inflammatory signaling, and vascular-cell activity, potentially contributing to the dyslipidemia, endothelial dysfunction, and progression of atherosclerosis observed after menopause. This hypothesis should not be interpreted as establishing FSH as an independent causal mediator in CCS: elevated FSH is tightly coupled to ovarian aging, declining estradiol concentrations, age, adiposity, and metabolic change, making residual confounding and collinearity unavoidable in existing studies. FSH is therefore best placed within the sex-hormone axis as a marker of menopausal neuroendocrine transition and a possible mechanistic co-modifier of cardiovascular risk, rather than as a validated stand-alone therapeutic target. Whether FSH adds clinically meaningful prognostic information beyond menopausal status, age, conventional risk factors, estradiol, and cardiometabolic variables remains unknown. No FSH threshold has been validated for CCS risk stratification, and no trial has tested whether an FSH-guided intervention improves angina, coronary microvascular dysfunction, atherosclerotic progression, or cardiovascular outcomes.

### 4.3. Hormone Therapy and Vascular Events

Menopausal hormone therapy has generated extensive—and sometimes conflicting—cardiovascular data. The Women’s Health Initiative randomized trials in predominantly older postmenopausal women did not demonstrate a reduction in coronary events and in some cases showed early increases in MI and stroke, particularly when therapy was started long after menopause or in women with established atherosclerosis [37]. Subsequent analyses support a “timing hypothesis”, whereby hormone therapy started in younger, recently menopausal women without advanced plaque may be neutral or modestly favorable, while late initiation in the presence of significant atherosclerotic burden may destabilize plaque and enhance thrombosis [36,37]. Formulation matters: transdermal estradiol regimens appear to carry a lower thrombotic risk than oral synthetic combinations, and the type of progestogen can independently influence endothelial and metabolic effects [37]. Non-hormonal alternatives such as phytoestrogens have been evaluated in randomized trials of postmenopausal women with limited effects on body composition and ambiguous cardiovascular benefit and should not currently be considered a substitute for evidence-based cardiovascular therapy [45].

In hypogonadal men with CAD, small randomized and observational studies suggest that testosterone replacement can improve exercise capacity, angina threshold, muscle strength, mood, and metabolic profile [39,43]. Concerns persist, however, about pro-thrombotic and pro-atherogenic effects of exogenous androgens, particularly at higher doses or in patients with polycythemia, sleep, or uncontrolled cardiovascular risk factors [39]. Menopausal hormone therapy and testosterone therapy should be considered only for appropriately established endocrine indications and individualized clinical circumstances. They should not be presented as therapies for the prevention or treatment of chronic coronary syndromes (CCS). Any discussion of these treatments should emphasize cardiovascular risk assessment, contraindications, and shared decision-making within the relevant endocrine or menopause-care framework.

## 5. Thyroid and Cardiometabolic Hormones in CCS

### 5.1. Thyroid Hormones

Thyroid hormones exert pervasive effects on cardiovascular structure and function, making thyroid status a critical modifier of CCS biology. Triiodothyronine (T3) and thyroxine (T4) modulate heart rate, myocardial contractility, systemic vascular resistance, lipid metabolism, and coagulation, so that even subclinical hypo- or hyperthyroidism can influence long-term cardiovascular risk [19,46]. In post-MI and established CAD populations, low-T3 syndrome and subclinical hypothyroidism predict higher rates of cardiovascular events, HF, and mortality, suggesting that impaired thyroid signaling marks a vulnerable cardiometabolic state [47,48].

Contemporary studies have specifically examined thyroid status in CCS and post-PCI populations. Subclinical hypothyroidism is associated with altered coagulation parameters and greater coronary disease severity [48], while a large retrospective cohort study found that thyroid dysfunction after PCI was associated with adverse cardiac remodeling and worse functional outcomes [30]. Beyond absolute hormone levels, indices of tissue-level thyroid hormone sensitivity have been independently associated with arrhythmia risk in coronary heart disease, indicating that the cardiac response to thyroid hormone—not only its circulating concentration—matters in CCS [49]. These observations argue for systematic thyroid assessment in CCS care, although robust interventional data on thyroid hormone supplementation or analogue therapy in CCS remain scarce [30,46,48]. These associations suggest that thyroid dysfunction may act both as a biological contributor to adverse remodeling and as a marker of underlying cardiovascular disease severity. Whether correcting subclinical thyroid abnormalities improves outcomes specifically in CCS populations remains uncertain, and interventional evidence is currently insufficient to support routine thyroid-targeted therapy solely for CCS risk modification. Levothyroxine is standard treatment for overt hypothyroidism and may be indicated in selected conventional endocrine settings. However, thyroid-hormone replacement should not be initiated solely to improve chronic coronary syndrome (CCS) outcomes, as evidence for such a strategy remains unproven. In patients with CCS, treatment decisions should follow established endocrine indications, with cautious dose initiation and titration where clinically appropriate.

### 5.2. Insulin, Incretins, Adipokines, and Obesity-Related Hormones

Chronic CAD and CCS frequently coexist with insulin resistance, type 2 diabetes mellitus, and obesity—clinical states defined by complex hormonal dysregulation [3,9]. Hyperinsulinemia, impaired incretin signaling, elevated leptin, reduced adiponectin, and altered levels of other adipokines define a neuroendocrine milieu that promotes atherosclerosis and microvascular dysfunction [9,23]. These hormonal disturbances drive endothelial dysfunction, oxidative stress, chronic low-grade inflammation, pro-thrombotic changes, and plaque vulnerability, amplifying residual risk beyond what can be explained by hyperglycemia alone [5,23,42].

Adipose tissue functions as a large endocrine organ secreting numerous adipokines with diverse cardiovascular actions [9]. Adiponectin is generally anti-inflammatory, insulin-sensitizing, and vasculoprotective, whereas leptin and pro-inflammatory adipokines rise with visceral obesity and contribute to sympathetic activation, endothelial dysfunction, impaired nitric-oxide signaling, and coronary microvascular impairment [9,23]. Epicardial and perivascular adipose depots are particularly relevant in CCS because their paracrine signaling directly modulates coronary endothelial function, vasomotor tone, and local inflammatory recruitment [9,23].

### 5.3. The Cardiometabolic CCS Phenotype

These observations support the concept of a distinct cardiometabolic CCS phenotype, characterized by obesity, insulin resistance or diabetes, non-alcoholic fatty liver disease, and high activation of multiple metabolic and adipose-derived hormonal axes [9,23]. In such patients, coronary disease is intertwined with systemic inflammation, endothelial and microvascular dysfunction, and adverse ventricular remodeling, driven at least as much by hormonal signals as by epicardial stenosis severity [3,23]. Clinically, this phenotype often presents with diffuse atherosclerosis, microvascular angina, HF with preserved ejection fraction (HFpEF), and a high burden of residual risk despite standard therapies [1,3].

Importantly, persistent low-grade inflammation appears to define a subgroup of CCS patients with a worse prognosis. Insights from the REAL-CAD study indicate that elevated inflammatory markers identify CCS patients at higher residual risk and may modify responses to several pharmacological therapies, including statins and neurohormonal modulators [5]. Integrating inflammatory phenotypes with hormonal profiling may therefore be informative for prognostic stratification and treatment selection. Although statins are not hormone-targeting agents in the conventional sense, their pleiotropic effects are relevant to the endocrine-inflammatory framework proposed here. Beyond LDL-cholesterol lowering, statins exert anti-inflammatory, antioxidant, endothelial-protective, and plaque-stabilizing actions that may reduce residual risk in CCS and interact with pathways linked to adipokine imbalance, RAAS activation, and persistent vascular inflammation. For this reason, any future phenotype-guided approach in CCS must be viewed as complementary to, and not substitutive of, optimal lipid-lowering therapy [50,51].

## 6. Hormone-Targeting Therapies in CCS

Hormone-modulating therapies occupy a central position in contemporary CCS management, yet their actions are often framed purely in hemodynamic terms rather than as interventions on specific hormonal axes [4,52]. A hormone-centric view highlights how RAAS inhibitors, beta-blockers, mineralocorticoid receptor antagonists (MRAs), sex-hormone interventions, thyroid-modulating drugs, and cardiometabolic agents (GLP-1 receptor agonists and SGLT2 inhibitors) interact with distinct neurohormonal and endocrine phenotypes [4,53,54]. This perspective supports a more deliberate alignment between hormonal profiles and therapeutic choices in CCS.

The evidence base underlying hormonal modulation in coronary disease is heterogeneous with respect to source population and clinical context. For clarity, we distinguish evidence directly obtained in contemporary CCS populations, evidence from broader stable CAD cohorts that may not fulfil current CCS definitions, evidence from post-MI or post-ACS populations, and evidence from HF cohorts with concomitant ischemic heart disease. These categories should not be considered interchangeable. In particular, apply therapies with established benefit in HF or post-MI populations to patients with CCS only when the corresponding HF, ventricular dysfunction, recent infarction, diabetes, kidney disease, or other guideline-supported indication is present. Unless specifically stated otherwise, extrapolation from these settings to stable CCS without HF or recent ACS should be regarded as indirect and hypothesis-generating.

### 6.1. RAAS Inhibitors

ACE inhibitors and ARBs are foundational in chronic CAD and CCS, traditionally justified by their effects on blood pressure, afterload, and post-MI ventricular remodeling [4,52]. A large body of experimental and clinical evidence shows that these drugs also exert anti-atherogenic, anti-inflammatory, and anti-fibrotic actions, particularly relevant to CCS [6,21]. By reducing angiotensin-II and aldosterone activity, RAAS inhibitors improve endothelial function, decrease oxidative stress, downregulate adhesion molecules, and attenuate smooth-muscle proliferation and matrix deposition [6,21]. In stable coronary populations, ACE inhibitors reduce MACE even in patients without overt HF, although the absolute benefit is smaller in lower-risk cohorts on intensive background therapy [24,25,26].

Angiotensin receptor–neprilysin inhibitors (ARNI), which enhance natriuretic-peptide signaling alongside RAAS blockade, have demonstrated benefits in HF with reduced ejection fraction; in HFpEF the PARAGON-HF trial (*n* = 4822) narrowly missed its primary endpoint (rate ratio 0.87; 95% CI 0.75–1.01), and data specifically in CCS without HF remain limited and largely extrapolated from HF populations [55].

In CCS patients with advanced CKD—a population at the intersection of RAAS overactivity and concerns about AKI/hyperkaliemia—ACEI/ARB therapy is associated with lower total and cardiovascular mortality, supporting cautious continuation rather than withdrawal in this high-risk group [26,27]. Optimal blood-pressure targets in CCS sit within this same RAAS-aware framework: contemporary guidance on hypertension management in CCS emphasizes both progression and onset reduction, integrating neurohormonal-axis considerations with conventional pressure targets [56].

### 6.2. Beta-Blockers and Sympathetic Modulation

Beta-blockers are traditionally used in CCS for symptom relief and post-MI secondary prevention based on heart-rate reduction, lower myocardial oxygen demand, and antiarrhythmic effects [4,57]. Their primary action, however, is modulation of the sympathetic–adrenal axis, which in CCS contributes to ischemia, arrhythmia, and vascular dysfunction [10,13,31]. By antagonizing β-adrenergic receptors, these agents blunt catecholamine-mediated tachycardia, reduce blood-pressure lability, limit calcium overload, and suppress arrhythmic triggers, stabilizing the ischemic substrate [10,13].

The contemporary evidence base for beta-blockers in CCS is, however, more nuanced than previously assumed. The recent REBOOT trial—a landmark analysis including 8438 patients in the acute phase and 7783 in the chronic phase of MI without reduced ejection fraction (LVEF > 40%)—found no reduction in the composite of all-cause death, non-fatal reinfarction, or HF hospitalization with beta-blocker therapy in either phase; notably, higher doses in the chronic phase were associated with worse outcomes [58]. The independent REDUCE-AMI trial (*n* = 5020) likewise found no benefit of beta-blockers after myocardial infarction with preserved ejection fraction (LVEF ≥ 50%; hazard ratio 0.96, 95% CI 0.79–1.16), whereas a pooled individual-patient analysis suggested possible residual benefit only in the mildly reduced range (LVEF 40–49%) [59]. Other large observational studies in stable ischemic heart disease and post-PCI CCS show variable findings, with benefits most apparent in subgroups defined by adequate heart-rate control, recent infarction, or impaired left-ventricular function [60,61,62,63,64]. Systematic reviews and meta-analyses in stable CAD without reduced ejection fraction suggest modest benefits at best, with substantial heterogeneity across studies [62,63].

Beta-blocker therapy in CCS should be presented as more nuanced in the contemporary era, particularly considering the REBOOT trial and recent observational data. In REBOOT, beta-blockers did not reduce the composite of all-cause death, non-fatal reinfarction, or heart-failure hospitalization in patients with acute or chronic MI and preserved ejection fraction, and higher doses in the chronic phase were associated with worse outcomes, suggesting that routine long-term use in all CCS patients without LV dysfunction may be unjustified. Conversely, several registries and cohort studies indicate that selected subgroups—such as patients with recent MI, impaired systolic function, tachycardia, or arrhythmic risk—may still derive prognostic benefit, particularly when adequate heart-rate control is achieved. Taken together, these findings support a shift from blanket, indefinite beta-blocker prescription in CCS toward individualized use focused on symptom control, clearly defined high-risk phenotypes, and microvascular or vasomotor indications, while also encouraging deprescribing or dose reduction in low-risk, asymptomatic patients with preserved ejection fraction.

These data argue for a shift from universal to selective beta-blocker prescription in CCS. In microvascular angina and INOCA, beta-blockers with vasodilatory or endothelium-protective properties (nebivolol, carvedilol) may offer additional benefits on coronary flow reserve, although the evidence base remains predominantly from small mechanistic studies [14,23,57]. Importantly, sympathetic over-activity may persist despite standard beta-blocker therapy in some patients with CCS and preserved ejection fraction, suggesting that conventional dosing strategies are sometimes inadequate to fully neutralize adrenergic drive [31].

### 6.3. Mineralocorticoid Receptor Antagonists: Potential Relevance in CCS

Evidence for MRAs in CCS without overt heart failure remains limited, and most available data are extrapolated from heart failure, chronic kidney disease, or mixed high-risk cardiovascular populations. No randomized trial has established mineralocorticoid receptor antagonism as a CCS-directed therapy in patients without HF, CKD, diabetes-related kidney disease, resistant hypertension, or another recognized indication.

MRAs such as spironolactone and eplerenone are proven therapies in HF with reduced ejection fraction (HFrEF), primarily through antifibrotic, natriuretic, and anti-remodeling actions that counter aldosterone excess [12,13]. In CCS without overt HF, the evidence is more limited and largely derived from small trials or post hoc analyses. Available data suggest that MRAs improve markers of endothelial function, reduce arterial stiffness, and decrease ventricular fibrosis surrogate markers in high-risk coronary patients, especially those with diabetes, CKD, or left-ventricular hypertrophy [12,28]. More recently, the non-steroidal MRA finerenone reduced the composite of cardiovascular death and worsening heart-failure events in heart failure with mildly reduced or preserved ejection fraction in FINEARTS-HF (*n* = 6001; ≈16% relative reduction, rate ratio 0.84), and lowered the primary kidney composite (hazard ratio 0.82) in type-2-diabetic chronic kidney disease in FIDELIO-DKD (*n* = 5734)—populations that overlap closely with the aldosterone-rich, CKD-associated CCS phenotype defined here [65,66]. Combined with a careful potassium and renal monitoring strategy, MRAs may therefore represent a rational extension of neurohormonal blockade in selected aldosterone-rich CCS phenotypes. Accordingly, MRAs should currently be viewed as biologically plausible adjuncts for selected CCS patients with overlapping HF, CKD, diabetes, or aldosterone-rich cardiometabolic profiles, rather than as established CCS-directed therapy. Finerenone should be discussed as a therapy with proven cardiorenal benefit in CKD associated with type 2 diabetes and in selected HF populations, whose benefit may be relevant to CCS patients with these comorbidities; it should not be presented as treatment for an ‘aldosterone-rich CCS phenotype’.

Mineralocorticoid receptor antagonists are established therapies when guideline-supported indications are present, including selected patients with heart failure, chronic kidney disease, hypertension, or type 2 diabetes with CKD. They should not, however, be presented as CCS-directed therapy in patients without these established indications. Their use should therefore be guided by the relevant comorbidity, renal function, serum potassium, and concomitant medical therapy.

### 6.4. Sex- and Thyroid-Hormone Interventions

In hypogonadal men with CAD, small randomized and observational studies suggest that testosterone replacement may improve exercise capacity, angina threshold, lean mass, and metabolic parameters [39,43]. Pro-thrombotic and pro-atherogenic effects of exogenous androgens at higher doses or in unstable patients remain a concern [39]. The randomized, placebo-controlled TRAVERSE trial (*n* = 5204 middle-aged and older men with hypogonadism and cardiovascular disease or risk factors) found no increase in major adverse cardiovascular events with testosterone replacement but a higher incidence of atrial fibrillation, pulmonary embolism, and acute kidney injury; testosterone should therefore be reserved for the treatment of symptomatic hypogonadism rather than used as a cardiovascular intervention [67]. In women with CCS, menopausal hormone therapy must be considered within the framework of the timing hypothesis and the individual atherosclerotic burden, with early transdermal regimens appearing safer than late oral initiation in advanced vascular disease [36,37].

Levothyroxine remains standard therapy for overt hypothyroidism and is sometimes prescribed in subclinical hypothyroidism when thyrotropin (TSH) is markedly elevated or when symptoms are significant [46,48]. Observational data linking subclinical hypothyroidism and low-T3 syndrome to adverse outcomes in CAD raise the possibility that optimizing thyroid status could improve long-term CCS trajectories [30,47,48]. Small experimental and pilot studies have evaluated low-dose T3 or thyroid hormone analogs in ischemic heart disease, with signals of improved ventricular function and reduced remodeling, but these findings remain preliminary [46,47].

### 6.5. Cardiometabolic Biologics: GLP-1 Receptor Agonists and SGLT2 Inhibitors

GLP-1 receptor agonists and SGLT2 inhibitors exemplify a growing class of biologically active therapies whose cardiovascular benefits are tightly linked to endocrine and metabolic actions. GLP-1 receptor agonists reduce MACE in patients with type 2 diabetes and established or high-risk CAD (e.g., liraglutide in LEADER, *n* = 9340; hazard ratio 0.87), with mechanisms extending beyond glucose lowering to include weight loss, blood-pressure reduction, improved endothelial function, and favourable effects on inflammation and adipokines [68]. SGLT2 inhibitors reduce HF hospitalization and cardiovascular mortality across broad cardiometabolic populations; in EMPA-REG OUTCOME (*n* = 7020) empagliflozin reduced three-point MACE by 14% (hazard ratio 0.86) and cardiovascular death by 38% [69,70]. The VERTIS CV trial (*n* = 8246; MACE hazard ratio 0.97, non-inferior to placebo) demonstrated that the cardiovascular and renal benefits of ertugliflozin were consistent regardless of baseline use of RAAS inhibitors, diuretics, or mineralocorticoid receptor antagonists, supporting additive rather than redundant effects of SGLT2 inhibition added to standard neurohormonal therapy [71]. Viewed as endocrine modulators, these drug classes alter incretin signaling, insulin–glucagon balance, natriuretic responses, adipokines, and, indirectly, RAAS and sympathetic tone [9,68,69,70].

A growing body of data supports low-grade inflammatory signaling via the IL-1β/IL-6 axis and the NLRP3 inflammasome as a key driver of residual cardiovascular risk in CCS, closely intertwined with classical neurohormonal and adipokine pathways. IL-1/IL-6–mediated inflammation amplifies RAAS and sympathetic activation, promotes endothelial dysfunction, and modulates adipose tissue cytokine output, thereby reinforcing the cardiometabolic CCS phenotype. Conversely, adipokine imbalance and neurohormonal stress can prime NLRP3 activation and sustain chronic vascular inflammation, underscoring a bidirectional endocrine–immune crosstalk. Although agents such as canakinumab and colchicine are not “hormones” in the traditional sense, they exemplify biologically targeted interventions that act on this inflammatory axis and have demonstrated reductions in ischemic events in high-risk atherosclerotic populations that overlap with contemporary CCS cohorts. These therapies therefore fit conceptually within a broader endocrine–immune framework, alongside RAAS inhibitors, GLP-1 receptor agonists, and SGLT2 inhibitors, as potential tools to modulate residual risk beyond anatomy.

### 6.6. Combined and Multi-Pathway Modulation

Combined pathway modulation is already common in CCS through the concurrent use of lipid-lowering, antithrombotic, RAAS-targeting, antianginal, and cardiometabolic therapies; however, direct evidence supporting combination strategies selected specifically by hormonal phenotype in CCS remains lacking. The most consistent signal for combined hormonal modulation in chronic ischemic populations comes from HF cohorts. A registry-based analysis of patients with chronic HF and mildly reduced or preserved ejection fraction showed that triple neuro-humoral modulation with beta-blocker, RAAS inhibitor, and MRA was associated with significantly better outcomes than no or single therapy (HR ≈ 0.70 in both HFmrEF and HFpEF), whereas double modulation alone did not show benefit, suggesting a threshold effect that requires comprehensive multi-pathway blockade [40]. A systematic review of beta-blockers alone versus combined with ACE inhibitors in chronic HF reported synergistic effects of combination therapy on mortality and HF hospitalization, with complementary mechanisms on neurohormonal activation, cardiac remodeling, and hemodynamics [64]. Fixed-dose combinations of ACE inhibitors and beta-blockers have been advocated by recent consensus documents for hypertensive patients at high cardiovascular risk, based on improved adherence, simplified regimens, and complementary pharmacology [53,54].

Across CCS more broadly, combined multi-pathway modulation aligned with the dominant hormonal phenotype appears more rational than uniform single-class escalation. The T.O.S.CA. Registry data showed that the presence of multiple hormonal and metabolic deficiencies (low T3, low IGF-1/GH, low testosterone, insulin resistance) predicts worse outcomes in HF patients, highlighting the prognostic importance of comprehensive endocrine assessment and supporting integrated hormonal management strategies in vulnerable populations [72]. Coexisting hormone-modulated conditions such as anemia further compound prognosis in chronic HF and demand integrated work-up [73]. Even in very old patients (≥80 years) with chronic HF, neurohormonal modulation retained its protective effect, with adjusted hazard ratios of 0.59 for both beta-blockers and RAAS inhibitors, suggesting that age and polypharmacy concerns should not preclude neurohormonal therapy in carefully selected patients [74]. Thus, evidence from HF supports the biological plausibility of multi-axis modulation, but in CCS, this concept remains hypothesis-generating and requires prospective validation. Most data on this strategy derive from HF cohorts with reduced or preserved EF, and extrapolation to CCS patients without HF or recent acute events should be undertaken with caution. In the present review, these therapies are discussed as potential options for CCS patients who also meet HF indications, rather than as treatments validated specifically for CCS without HF.

### 6.7. Toward a Hormonal Treatment Map for CCS

**Figure 2:**
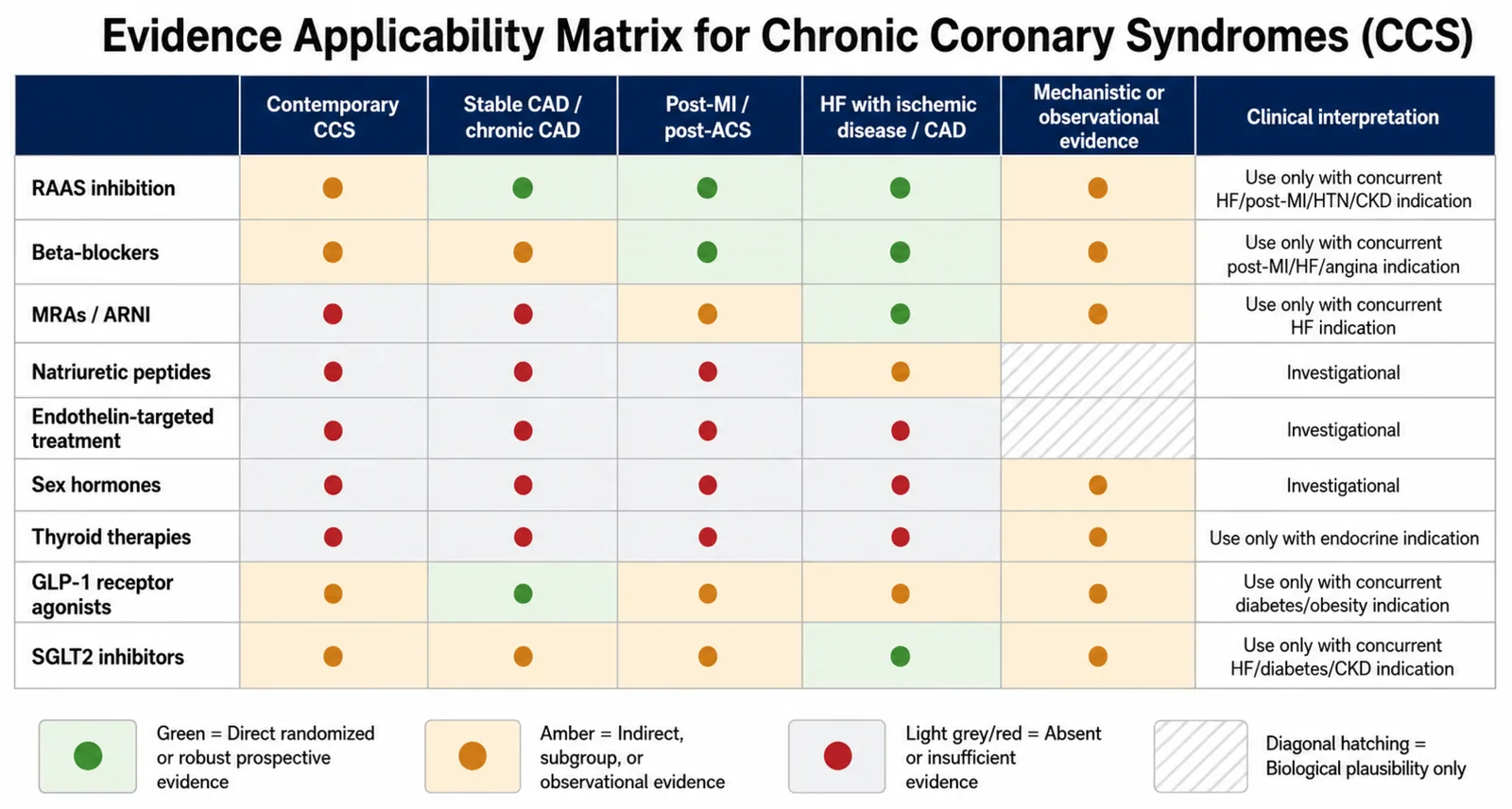
Evidence-applicability matrix for pharmacological and endocrine-metabolic interventions in contemporary CCS. The matrix differentiates evidence that is directly applicable to contemporary CCS from evidence derived predominantly from CCS, post-MI/ACS, HF with concomitant ischemic disease or CAD, and mechanistic or observational studies. Green cells indicate direct randomized-trial evidence or robust prospective evidence; amber cells indicate indirect evidence, subgroup findings, or observational support; light grey/red cells indicate absent or insufficient evidence; hatched cells denote biological plausibility without established clinical efficacy. The final column translates the evidence base into clinical interpretation: CCS-directed treatment, use only when a concurrent evidence-based indication is present (e.g., HF, recent MI, diabetes, chronic kidney disease, hypertension, obesity, or endocrine disease), or investigational use. This framework highlights the differing degree of extrapolation required when applying therapies across the CCS spectrum.

Synthesizing the above evidence supports a conceptual hormonal treatment map for CCS, in which specific therapies are preferentially aligned with dominant hormonal phenotypes (Table 1, Supplementary Table S3 and Figure 2). RAAS inhibitors and MRAs target RAAS-driven and aldosterone-rich CCS phenotypes; beta-blockers address sympathetic-dominant states (with attention to dose, ejection fraction, and microvascular involvement); GLP-1 receptor agonists and SGLT2 inhibitors are particularly suited to cardiometabolic-dominant CCS; and sex- and thyroid-hormone interventions may be relevant in carefully selected sex- or thyroid-modulated phenotypes [4,6,9]. Current guidelines reflect this logic only partially, treating hormonal factors mostly as comorbidities rather than integral components of CCS pathophysiology and treatment algorithms [1,2]. The treatment map presented in Table 1, Supplementary Table S3 and Figure 2 is intended as a conceptual alignment between putative hormonal phenotypes and existing or emerging therapies, not as a prescriptive schema for clinical decision-making. The validity, reproducibility, and prognostic utility of these phenotypes, as well as the incremental benefit of phenotype-matched therapy over standard guideline-based care, must be tested in prospective CCS studies before routine implementation.

The proposed framework is intended as a research-oriented tool to support patient stratification, mechanistic hypothesis generation, and the design and enrollment of future clinical trials. It should not be interpreted as a validated clinical decision algorithm or as a substitute for guideline-directed diagnostic assessment and treatment. Prospective studies are needed to determine whether endocrine and neurohormonal profiling can identify actionable subgroups and improve clinical outcomes.

## 7. Discussion and Future Directions

### 7.1. Conceptual Gaps

Despite major advances in imaging and revascularization, CCS is still predominantly conceptualized as an anatomical or ischemic burden problem, with treatments focused on luminal narrowing, plaque burden, and flow-limiting lesions [1]. Hormonal axes—neurohormonal, sex, thyroid, and cardiometabolic—are usually treated as parallel comorbidities rather than as integral determinants of CCS phenotypes, symptom burden, and residual risk [3,9]. No unified hormone-centric CCS model systematically integrates RAAS, sympathetic activation, natriuretic peptides, ET-1, sex steroids, thyroid hormones, insulin, incretins, and adipokines across the spectrum of CCS presentations [6,7,9]. Current evidence highlights substantial gaps in biomarker-based phenotyping of CCS, particularly in integrating neurohormonal, sex-hormone, thyroid, and cardiometabolic markers into reproducible, clinically usable profiles. Very few studies or randomized trials in stable CCS prospectively stratify patients by hormonal or endocrine status, so it remains unknown whether treatment effects differ meaningfully across such biologically defined subgroups or whether biomarker-guided algorithms can improve risk prediction and therapeutic response beyond conventional clinical variables. Inflammation interacts bidirectionally with the hormonal axes considered in this framework: RAAS and sympathetic activation, adipokine imbalance, and thyroid dysfunction can sustain pro-inflammatory signaling, while chronic low-grade inflammation enhances neurohormonal activation and cardiometabolic stress. From a practical standpoint, inflammation should be regarded as a complementary domain that overlaps with multiple hormonal phenotypes rather than as a separate axis; inflammatory biomarkers and anti-inflammatory therapies may refine risk stratification within RAAS-dominant, cardiometabolic, or microvascular CCS subsets without altering the endocrine focus of the present classification.

### 7.2. Methodological Gaps

Methodological limitations amplify these conceptual gaps. Definitions of CCS versus chronic CAD remain heterogeneous; many trials still enroll broad chronic-CAD populations without distinguishing obstructive from non-obstructive disease or characterizing microvascular function [75]. Recent efforts to standardize CAD domains and to define delayed or missed CAD diagnosis illustrate the need for a clearer separation between acute coronary syndromes and CCS, and for more precise CCS sub-phenotypes [75]. Hormonal data within cardiovascular trials are often sparse, fragmented, or limited to single markers, and longitudinal sampling is uncommon, making it difficult to relate temporal changes in hormonal status to CCS progression, residual risk, or therapeutic response [3,42]. The recent literature also suffers from the under-representation of women and from few interventional trials of endocrine therapies with cardiovascular endpoints [37,41].

### 7.3. Proposed Research Agenda

The next decade should focus on connecting biology to CCS phenotypes and to outcome-modifying therapies. Table 2 summarizes priority research domains. Particularly informative would be prospective CCS cohorts combining multi-hormone profiling with detailed coronary physiology, vasomotor testing, and imaging; well-powered randomized trials of endothelin antagonism (e.g., zibotentan) in microvascular angina; pragmatic trials revisiting beta-blocker indications and dosing in contemporary CCS without reduced ejection fraction; and trials of integrated hormonal management strategies in patients with multiple hormonal deficiencies or pronounced cardiometabolic dysregulation.

In this review, hormonal profiles are conceptual research constructs rather than validated clinical phenotypes. At present, no consensus thresholds, reproducible clustering algorithm, or prospective biomarker-stratified trial supports assignment of individual patients to a RAAS-dominant, aldosterone-associated, sex-hormone-related, thyroid-related, or cardiometabolic CCS category. The profiles in Table 2 should therefore be interpreted as overlapping biological patterns identified through clinical context, comorbidities, standard laboratory testing, imaging, and—where clinically indicated—selected endocrine measurements. They are not mutually exclusive, and no profile currently mandates a CCS-specific treatment beyond therapies indicated by contemporary CCS, hypertension, diabetes, CKD, HF, or endocrine guidelines. A central distinction is required between a hormonal signal that is associated with risk and a hormonal signal that is actionable. Across several endocrine axes in CCS, circulating levels or pathway surrogates have prognostic or mechanistic relevance, but evidence that treatment directed by those measurements improves symptoms or cardiovascular outcomes is absent. Accordingly, an abnormal hormonal signal should not be interpreted as an indication for hormone-targeted therapy unless treatment is independently supported by an established endocrine, renal, metabolic, or heart-failure indication. The labels RAAS-dominant, aldosterone-associated, cardiometabolic-dominant, thyroid-related, and sex-hormone-related describe overlapping biological hypotheses, not validated patient categories. No biomarker threshold, composite score, or clustering algorithm currently supports their assignment in individual patients. Evidence that a pathway is prognostic or mechanistically plausible should not be interpreted as evidence that pathway-directed treatment improves CCS outcomes.

### 7.4. Integrating Endocrine and Neurohormonal Profiling with Established CCS Phenotypes

Contemporary CCS should first be classified according to established clinical and pathophysiological profiles, including obstructive focal or diffuse epicardial coronary artery disease, post-myocardial infarction or post-revascularization CCS, ANOCA/INOCA, coronary microvascular dysfunction, vasospastic angina, and CCS with concomitant heart failure or cardiometabolic disease. These profiles differ in dominant mechanisms, diagnostic testing, baseline guideline-directed management, and expected treatment response.

The endocrine and neurohormonal framework proposed in this review is not intended to replace these categories. Instead, it represents an additional biological layer that may help explain heterogeneity in symptom burden, residual risk, microvascular dysfunction, ventricular remodelling, and response to therapies within each established CCS phenotype. For example, RAAS activation and aldosterone excess may be particularly relevant in diffuse atherosclerotic disease, hypertension, CKD, and post-MI remodelling; sympathetic predominance may contribute to tachycardia-associated angina, stress-related symptoms, and selected vasomotor or microvascular presentations; endothelin-related dysregulation may be especially pertinent to CMD and vasomotor disease; and adipokine, insulin, incretin, and inflammatory pathways may modify risk in obesity- and diabetes-associated CCS with HFpEF overlap.

Accordingly, the proposed model should be interpreted as a matrix rather than a replacement classification. A patient may have post-PCI obstructive CCS with diabetes, obesity, CKD, and HFpEF, in whom diffuse atherosclerosis, impaired coronary microvascular reserve, RAAS activation, adipose-related inflammation, and increased wall stress coexist. Conversely, a patient with ANOCA/INOCA may have a predominantly vasomotor, microvascular, autonomic, or endocrine-metabolic substrate despite the absence of flow-limiting epicardial disease. In both situations, clinical phenotype and standard guideline-based management remain primary; endocrine and neurohormonal profiling remains exploratory and should be used to formulate mechanistic hypotheses rather than to define a new stand-alone treatment algorithm.

Future studies should therefore prospectively evaluate whether endocrine and neurohormonal markers provide incremental prognostic or predictive value within predefined CCS profiles, rather than across unselected chronic CAD populations.

**Figure 3:**
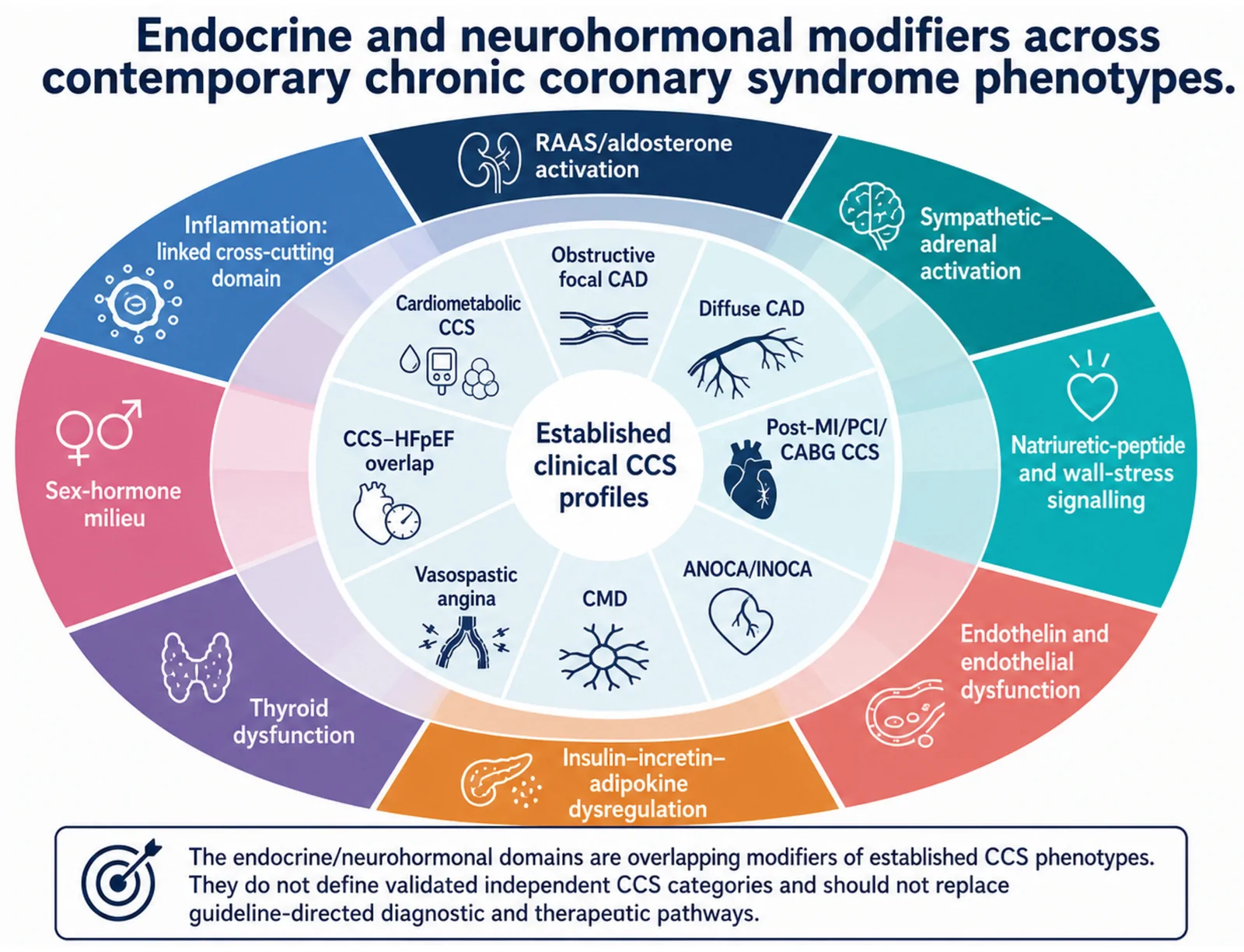
Endocrine and neurohormonal modifiers across contemporary chronic coronary syndrome phenotypes. The inner ring depicts clinically recognizable chronic coronary syndrome (CCS) profiles, spanning obstructive and diffuse coronary artery disease, prior myocardial infarction or revascularization, ANOCA/INOCA, coronary microvascular dysfunction, vasospastic angina, CCS–HFpEF overlap, and cardiometabolic CCS. The outer ring illustrates endocrine and neurohormonal domains that may interact with, amplify, or partly mediate symptom burden, vascular dysfunction, myocardial remodelling, and residual cardiovascular risk across these profiles. In particular, RAAS/aldosterone and sympathetic–adrenal activation, natriuretic-peptide/wall-stress signalling, endothelin-mediated endothelial dysfunction, insulin–incretin–adipokine dysregulation, thyroid dysfunction, sex-hormone milieu, and inflammation may coexist and exert overlapping effects rather than acting as mutually exclusive mechanisms. Contemporary CCS assessment recognizes that obstructive epicardial disease, microvascular dysfunction, and vasomotor abnormalities may overlap within individual patients. Abbreviations: ANOCA, angina with non-obstructive coronary arteries; CABG, coronary artery bypass grafting; CCS, chronic coronary syndrome; CMD, coronary microvascular dysfunction; HFpEF, heart failure with preserved ejection fraction; INOCA, ischaemia with non-obstructive coronary arteries; MI, myocardial infarction; PCI, percutaneous coronary intervention; RAAS, renin–angiotensin–aldosterone system.

This framework is therefore intended to complement established CCS phenotyping by linking clinical presentation to underlying endocrine and neurohormonal pathways that may become therapeutically actionable in the future. The central novel proposal of this review is a CCS classification based on dominant hormonal axes. In this framework, CCS is placed at the center of a network with four major hormonal domains: neurohormonal (RAAS/sympathetic/natriuretic peptide/ET-1), sex-hormone-modulated, cardiometabolic-dominant (insulin/adipokines/incretins), and thyroid-modulated CCS [6,9,19]. Each axis can predominate or coexist to varying degrees, shaping ischemia, plaque behavior, microvascular function, symptoms, and progression [14,23]. Aligning this classification with the treatment map outlined in Table 1 could enable more rational targeting of RAAS inhibitors, beta-blockers, MRAs, sex- and thyroid-hormone strategies, and cardiometabolic biologics to specific hormonal phenotypes rather than applying them uniformly across all CCS patients [4,40,58,71]. At present, these hormonal phenotypes cannot be considered validated clinical categories. In routine practice, they would most plausibly be inferred from a combination of conventional clinical features and accessible biological surrogates—such as diabetes/obesity status, thyroid function tests, natriuretic peptides, sex-hormone status in selected patients, renal function, blood pressure pattern, heart rate, and inflammatory markers—rather than from a single definitive biomarker. Considerable overlap between phenotypes should be expected, and in many patients the dominant mechanism may remain uncertain. Accordingly, the proposed classification should currently be regarded as a conceptual scaffold for research and clinical reasoning, not as a replacement for existing CCS algorithms. Supplementary Table S4 positions the hormonal-phenotype framework proposed here against the CCS phenotypes already recognized in clinical practice and in current guideline documents. Rather than advancing a parallel taxonomy, it asks a narrower question of each established phenotype: which endocrine and neurohormonal pathways are plausibly active, which of the assessments already indicated in routine care might capture them, and how far the supporting evidence extends. Three patterns are apparent. First, RAAS and sympathetic activation recur across almost every phenotype and are therefore of limited discriminative value; their presence identifies the disease rather than the subgroup. Second, the modifiers that are phenotype-specific—endothelin excess in coronary microvascular dysfunction, sex-hormone influences in ANOCA/INOCA and vasospastic angina, and incretin and adipokine dysregulation in cardiometabolic CCS—are precisely those for which interventional evidence is weakest, so the axes with the greatest discriminative potential are the least tested. Third, the phenotypes are not mutually exclusive, and individual patients frequently satisfy several categories simultaneously, which constrains any attempt to assign a single dominant hormonal signature. Accordingly, none of the modifiers listed has been validated as a treatment-selection criterion, and the table should be read as identifying where hormonal profiling might add information to existing phenotyping rather than as a basis for modifying guideline-directed management. The endocrine/neurohormonal domains shown are cross-cutting modifiers, not validated independent CCS categories; they should not replace guideline-directed diagnostic classification, endotype assessment, or therapeutic pathways (Figure 3).

### 7.5. Limitations

A major limitation of this work is its inherently narrative design: despite being informed by a structured literature search and explicit eligibility criteria, it does not include quantitative synthesis, formal risk-of-bias evaluation, or graded certainty of evidence, and thus cannot estimate the causal effects of specific hormonal interventions on CCS outcomes. The hormonal-phenotype framework and treatment map should therefore be viewed as a biologically motivated conceptual model, developed in part by extrapolating data from heart failure, acute coronary syndromes, and high-risk cardiovascular cohorts that may not fully reflect the spectrum of stable CCS. Several key domains—such as endothelin-directed therapies, testosterone replacement in men with CAD, menopausal hormone therapy in women with established CCS, and thyroid-hormone modulation—remain underpinned predominantly by small, observational, or mechanistic studies, limiting the robustness of any practice-oriented recommendations. Future research should focus on prospective, CCS-specific cohorts and adequately powered randomized trials that stratify patients according to predefined hormonal and cardiometabolic profiles, rigorously test phenotype-guided treatment strategies (for example, cardiometabolic-dominant CCS managed with GLP-1 receptor agonists and SGLT2 inhibitors, or neurohormonal-dominant CCS treated with tailored RAAS and sympathetic modulation), and incorporate multi-axis hormonal biomarkers alongside imaging and omics-based metrics. Validation of such phenotypes, including their reproducibility, prognostic value, and treatment responsiveness, will be essential before a biology-centric framework can be integrated into clinical pathways or guideline recommendations. A further limitation is that no validated biomarker panel currently distinguishes the proposed hormonal phenotypes with sufficient specificity to guide routine CCS treatment decisions, and the incremental prognostic or therapeutic value of these panels beyond established clinical phenotyping and comorbidity-based management remains unproven. A substantial proportion of the evidence underpinning this framework originates from HF, post-ACS, and high-risk vascular cohorts, and may not directly apply to stable CCS patients without HF or recent events. We have therefore consistently indicated where inferences are extrapolations rather than CCS-specific data, and we emphasize that the proposed phenotypes and treatment suggestions must be validated in dedicated CCS populations. We acknowledge that no formal risk-of-bias assessment, certainty-of-evidence grading, or quantitative meta-analysis was undertaken. This reflects the intentionally heterogeneous nature of the included literature, which spans mechanistic, observational, clinical, and hypothesis-generating evidence. Accordingly, the review should be interpreted as a conceptual synthesis rather than a systematic evaluation of treatment effects or causal associations. Its purpose is to propose an integrative framework and identify priorities for future targeted research.

## 8. Clinical Implications

The distinction between a hormonal-phenotype approach and standard comorbidity-based treatment lies mainly in biological emphasis rather than in a wholly different therapeutic armamentarium. Current practice tailors therapy according to diabetes, CKD, obesity, thyroid disease, menopausal status, or ventricular dysfunction; the proposed framework instead asks whether these comorbidities reflect a dominant endocrine or neurohormonal mechanism that also shapes ischemia, symptoms, and residual risk within CCS itself. At present, this distinction is conceptual rather than operational because validated biomarker-guided pathways are lacking. However, it may help generate testable hypotheses for trials that move beyond anatomy and comorbidity labels toward mechanism-enriched CCS subgroups. A crucial distinction must be made between hormonal signals associated with CCS risk and hormonal signals that are actionable treatment-selection markers. Across several axes, circulating hormone concentrations or pathway surrogates have mechanistic or prognostic relevance—for example, thyroid dysfunction or low-T3 states, sex-hormone and FSH-related changes, endothelin dysregulation, natriuretic peptide elevation, and adipokine abnormalities—but there is currently no evidence that assigning CCS therapy on the basis of these measurements improves symptoms or cardiovascular outcomes. No consensus thresholds, reproducible clustering method, or prospectively validated biomarker algorithm is available to classify an individual patient as RAAS-dominant, aldosterone-associated, thyroid-related, sex-hormone-related, or cardiometabolic-dominant. These profiles overlap substantially and should therefore be considered research constructs rather than clinical labels.

Conversely, where therapies that influence neurohormonal or cardiometabolic pathways have established outcome benefits, their use is generally determined by conventional clinical indications—such as hypertension, prior myocardial infarction, left-ventricular dysfunction or heart failure, diabetes, chronic kidney disease, and obesity—rather than by measurement of the relevant hormonal axis. Thus, RAAS inhibitors, beta-blockers, GLP-1 receptor agonists, and SGLT2 inhibitors should not be interpreted as evidence for a validated hormone-guided CCS strategy. For endocrine-directed interventions, including thyroid hormone replacement, testosterone therapy, menopausal hormone therapy, and endothelin receptor antagonism, current evidence does not support their use to improve CCS outcomes outside established endocrine indications or appropriately designed clinical trials. Future CCS-specific trials must prospectively define hormonal eligibility criteria and test whether biomarker- or phenotype-stratified treatment changes patient-centred outcomes before this framework can become clinically actionable. The proposed endocrine and neurohormonal framework is intended to complement—not replace—established CCS classification, diagnostic evaluation, and guideline-directed management. At present, no validated biomarker threshold, composite score, or reproducible algorithm permits assignment of an individual patient to a RAAS-dominant, aldosterone-associated, thyroid-related, sex-hormone-related, or cardiometabolic CCS phenotype. Accordingly, these constructs should not be used as stand-alone treatment-selection criteria.

1. Clinicians should ensure comprehensive implementation of guideline-directed CCS care, including lifestyle measures, lipid-lowering and antithrombotic therapy, antianginal treatment, blood-pressure control, and revascularization where indicated. RAAS inhibitors and beta-blockers should be prescribed according to established CCS and comorbidity-specific indications, including hypertension, prior myocardial infarction, left-ventricular dysfunction, heart failure, arrhythmia, or symptom control, rather than according to a presumed hormonal profile.
2. GLP-1 receptor agonists and SGLT2 inhibitors may be appropriate in CCS patients with established evidence-based indications such as type 2 diabetes, obesity, heart failure, and/or chronic kidney disease, in accordance with the relevant cardiovascular, diabetes, obesity, heart-failure, and kidney-disease guidelines. Their use should not currently be interpreted as phenotype-guided endocrine therapy for CCS in the absence of these indications.
3. Routine clinical assessment should identify and manage coexisting thyroid disease, obesity, diabetes, chronic kidney disease, menopausal symptoms, and confirmed hypogonadism when clinically indicated. Treatment of thyroid dysfunction, menopausal symptoms, or hypogonadism should follow established endocrine or menopause-care indications; it should not be initiated solely to modify CCS symptoms, coronary physiology, or cardiovascular outcomes.
4. Endothelin receptor antagonists, hormone replacement strategies intended to improve cardiovascular outcomes, thyroid hormone analogues, and multi-axis biomarker-guided treatment approaches remain investigational in CCS. These interventions should be restricted to clinical trials or individualized non-CCS indications, with appropriate specialist assessment.
5. The proposed framework may currently be useful only as a research-oriented lens for generating mechanistic hypotheses and designing CCS-specific studies. Its future clinical role depends on prospective validation of reproducible phenotype definitions, clinically usable thresholds, incremental prognostic value beyond standard assessment, and randomized evidence that phenotype-stratified therapy improves patient-centred outcomes.

## 9. Conclusions

Chronic coronary syndrome should be understood as a heterogeneous clinical condition in which anatomical coronary disease, ischemia, inflammation, vasomotor and microvascular dysfunction, cardiometabolic status, and neurohormonal/endocrine pathways interact. The evidence reviewed supports the clinical relevance of neurohormonal and endocrine disturbances as potential modifiers of symptoms, myocardial remodelling, and residual cardiovascular risk, but does not yet establish them as validated CCS phenotypes or treatment-selection criteria.

The hormonal-phenotype framework proposed in this review is therefore intended as a conceptual and hypothesis-generating complement to established guideline-based CCS classification and management, rather than as a new clinical decision algorithm. RAAS inhibition, beta-blockers, GLP-1 receptor agonists, SGLT2 inhibitors, and endocrine therapies should continue to be used according to their established CCS- or comorbidity-specific indications, not solely on the basis of a presumed hormonal profile. At present, hormonal profiling should not determine CCS treatment selection, since no phenotype-specific thresholds or biomarker-guided intervention trials have demonstrated improved clinical outcomes. Prospective CCS-specific studies are needed to determine whether reproducible endocrine and neurohormonal signatures provide incremental prognostic information and, crucially, identify patient subgroups in whom phenotype-guided interventions improve clinical outcomes.

## Figures and Tables

**Table 1:** Hormonal treatment map for CCS, aligning dominant hormonal axes with preferred therapeutic strategies, current supporting evidence, evidence type and clinical status. CCS = chronic coronary syndrome; CKD = chronic kidney disease; CMD = coronary microvascular dysfunction; EF = ejection fraction; HFmrEF/HFpEF = heart failure with mildly reduced/preserved ejection fraction; HRT = hormone replacement therapy; MASLD = metabolic dysfunction-associated steatotic liver disease; MRA = mineralocorticoid receptor antagonist; ARNI = angiotensin receptor–neprilysin inhibitor; INOCA/ANOCA = ischemia/angina with non-obstructive coronary arteries; FSH = follicle-stimulating hormone. This table is a hypothesis-generating conceptual model rather than a treatment recommendation. The two right-hand columns are provided precisely to prevent the table from being read as a treatment algorithm: they separate agents with randomized outcome evidence in CCS from those whose evidence derives from other populations, and they distinguish hormones that are prognostic markers from hormones that are actionable treatment targets. Critically, none of the therapies listed—including those with the strongest evidence—is currently prescribed on the basis of a measured hormonal value; no hormonal axis in this table has a validated diagnostic cutoff or a hormone-guided treatment strategy validated for CCS endpoints. The proposed hormonal categories are not validated clinical phenotypes and should not be used to assign CCS treatment outside established guideline indications. Biomarker abnormalities are currently interpreted as mechanistic or prognostic signals unless a CCS-specific, biomarker-stratified intervention trial has demonstrated clinical utility.

Hormonal Axis	Dominant CCS Phenotype	Preferred Hormone-Targeting Strategies	Supporting Evidence	Evidence Strength	Evidence Type	Clinical Status
**RAAS/aldosterone**	Obstructive and diffuse atherosclerotic CCS; post-MI; HFmrEF/HFpEF; CCS + CKD	ACE inhibitors, ARBs, MRAs; ARNI in selected HF overlap	[24,25,26,27,40,55]	RCT-supported (ACE inhibitors, ARBs); moderate for MRAs/finerenone	**ACE inhibitors/ARBs:** therapeutic evidence in CCS (HOPE, EUROPA in stable CAD)—benefit demonstrated in unselected populations and not contingent on measurement of renin, angiotensin II or aldosterone. **MRAs/finerenone/ARNI:** therapeutic evidence only from non-CCS populations (HFmrEF/HFpEF, CKD with type 2 diabetes). **Circulating aldosterone/renin as phenotype descriptors:** mechanistic and observational-prognostic.	**ACE inhibitors/ARBs:** established CCS management (guideline-recommended, prescribed on clinical indication rather than on hormonal profile). **MRAs/finerenone/ARNI:** potentially actionable for comorbidity-based care (coexisting HF, CKD or post-MI LV dysfunction); not established CCS-directed therapy in their absence. **Aldosterone-guided drug selection:** research only—no validated diagnostic cutoff.
**Sympathetic–adrenal**	Post-MI with reduced EF; tachycardia-dominant; arrhythmic CCS	Beta-blockers (selective use; consider vasodilatory β-blockers in CMD)	[57,58,60,61,62,63]	RCT-supported in reduced EF; neutral in preserved EF (REBOOT, REDUCE-AMI)	**Beta-blockers:** therapeutic evidence in CCS and post-MI populations, positive in reduced EF and neutral in preserved EF (REBOOT, REDUCE-AMI); indication defined by ejection fraction, heart rate and symptoms, not by catecholamine measurement. **Catecholamines, heart-rate variability, muscle sympathetic nerve activity:** mechanistic and observational-prognostic. **Vasodilatory β-blockers in CMD:** mechanistic, with small physiological studies only.	**Beta-blockers:** established CCS management for angina control and for post-MI reduced EF; currently under legitimate re-evaluation in preserved EF. **Sympathetic-biomarker-guided β-blocker selection or dosing:** research only. **Vasodilatory β-blockers in CMD:** investigational phenotype marker–based approach.
**Natriuretic/counter-regulatory**	CCS with subclinical remodelling, diastolic dysfunction, or early HFpEF	RAAS inhibitors; ARNI in HF; natriuretic-peptide-guided monitoring	[11,12,32,33,55]	Moderate; mainly observational/biomarker-based	**BNP/NT-proBNP in CCS:** observational-prognostic, with consistent and graded association with mortality and HF events (including PEACE). **ARNI:** therapeutic evidence only from non-CCS populations (symptomatic HF). **Natriuretic-peptide-guided titration:** tested in HF only; no CCS trial has randomized treatment to a natriuretic-peptide threshold.	**NT-proBNP measurement:** established for risk stratification and for detection of coexisting HF, i.e., potentially actionable for comorbidity-based care. **ARNI:** potentially actionable for comorbidity-based care (HF overlap) only. **Natriuretic-peptide-guided CCS treatment strategy:** research only.
**Endothelin**	Microvascular angina; INOCA/ANOCA; vasomotor CCS	ETA antagonism (investigational, e.g., PRIZE)	[34,35]	Mechanistic–hypothetical; investigational (PRIZE ongoing)	**ET-1 signalling:** mechanistic (vasoconstriction, endothelial dysfunction, fibrosis). **Circulating ET-1:** observational-prognostic in small cohorts. **ETA antagonism:** early-phase therapeutic evidence in a CCS-adjacent population (microvascular angina), with symptom/physiology endpoints and no outcome data.	**ET-1:** investigational phenotype marker—no validated diagnostic cutoff and no assay standardization for clinical use. **ETA antagonism:** research only; not part of any current CCS management pathway.
**Sex hormones**	Postmenopausal CCS; hypogonadal men with CAD; microvascular angina in women	Individualized HRT (timing hypothesis); selective testosterone use	[36,37,39,41,43]	Weak/extrapolated; observational and mechanistic	**Estrogen, testosterone, FSH:** mechanistic and observational-prognostic. **Menopausal hormone therapy:** therapeutic evidence only from non-CCS populations (menopausal symptom trials), with age- and timing-dependent effects and no CCS outcome trial. **Testosterone replacement:** therapeutic evidence only from non-CCS populations—a cardiovascular safety trial in symptomatic hypogonadism (TRAVERSE), not an efficacy trial for CCS endpoints. **FSH as a postmenopausal risk descriptor:** observational-prognostic only.	**Hormone replacement therapy:** potentially actionable for comorbidity-based care (menopausal symptoms, hypogonadism), prescribed on endocrine, not cardiological, indication. **Sex hormones as CCS treatment targets:** research only—no validated cutoff and no sex-hormone-guided CCS treatment strategy. **FSH:** investigational phenotype marker.
**Thyroid hormones**	Low-T3 syndrome; subclinical hypothyroidism; post-PCI remodelling	Levothyroxine when indicated; investigational T3/analogues	[30,46,47,48,49]	Observational and mechanistic; no outcome RCTs	**Thyroid hormone action on the myocardium and vasculature:** mechanistic. **Low-T3 syndrome and subclinical hypothyroidism:** observational-prognostic, and at least partly a marker of illness severity rather than a causal driver. **Levothyroxine:** therapeutic evidence only from non-CCS populations (hypothyroidism), neutral for cardiovascular outcomes in subclinical disease; no CCS outcome RCT. **T3 and thyromimetic analogues:** mechanistic and small physiological studies.	**Levothyroxine for overt hypothyroidism:** potentially actionable for comorbidity-based care. **Low T3:** investigational phenotype marker—prognostically informative but with no validated treatment threshold. **T3/analogue administration in CCS:** research only.
**Cardiometabolic/adipose**	Diabetes-, obesity-, MASLD-associated CCS; HFpEF overlap	GLP-1 receptor agonists; SGLT2 inhibitors; weight management	[9,68,69,70,71]	RCT-supported (GLP-1 receptor agonists, SGLT2 inhibitors)	**GLP-1 receptor agonists and SGLT2 inhibitors:** therapeutic evidence from defined cardiometabolic populations (type 2 diabetes, obesity, HF, CKD), many of whom have established CAD; eligibility is defined by the comorbidity and not contingent on measurement of any hormonal biomarker. **Insulin resistance:** mechanistic and observational-prognostic. **Adipokines (leptin, adiponectin, resistin):** mechanistic and observational-prognostic; no interventional data.	**GLP-1 receptor agonists/SGLT2 inhibitors:** established for cardiovascular risk reduction and therefore potentially actionable for comorbidity-based care in CCS patients with diabetes, obesity, HF or CKD; not established CCS-directed therapy in their absence. **Adipokine- or insulin-guided therapy selection:** research only—no validated diagnostic cutoff.

*Category definitions applying to both tables:* Evidence type—Mechanistic: preclinical, physiological or pathway-level data only. Evidence type—Observational-prognostic: the hormone or biomarker is associated with outcome in human cohorts, without evidence that modifying it changes prognosis. Evidence type—Therapeutic evidence in CCS: randomized outcome data obtained in stable CAD/CCS populations. Evidence type—Therapeutic evidence only from non-CCS populations: randomized outcome data confined to heart failure, chronic kidney disease, type 2 diabetes, obesity or acute coronary populations, and extrapolated to CCS. Clinical status—Established CCS management: guideline-endorsed component of contemporary CCS care. Clinical status—Potentially actionable for comorbidity-based care: indicated when a specific coexisting condition is present, on the basis of that condition rather than of CCS itself. Clinical status—Investigational phenotype marker: biologically plausible and measurable, under active evaluation, but without a validated diagnostic cutoff. Clinical status—Research only: neither a validated assay threshold nor a hormone-guided treatment strategy exists.

**Table 2:** Priority research agenda for hormonal modulation in CCS, with the current evidence type and clinical status of each domain. CCS = chronic coronary syndrome; EF = ejection fraction; ET-1 = endothelin-1; FSH = follicle-stimulating hormone; HFmrEF/HFpEF = heart failure with mildly reduced/preserved ejection fraction; OCT/IVUS = optical coherence tomography/intravascular ultrasound; SGLT2 = sodium–glucose cotransporter-2; ARNI = angiotensin receptor–neprilysin inhibitor. In this table, the two added columns describe the evidence and clinical standing that currently underpin each research domain, not the status the proposed studies would confer; the proposals themselves are, by definition, not yet actionable. Category definitions are as given beneath Table 1.

Research Domain	Priority Proposal	Rationale	Evidence Type (Current)	Clinical Status (Current)
**Observational**	Prospective CCS cohorts with multi-hormone profiling: RAAS, autonomic indices, natriuretic peptides, sex hormones, thyroid panels, insulin/GLP-1, and adipokines	Define phenotype-specific hormonal signatures and link them to ischemia, imaging, and outcomes [3,9,42].	Observational-prognostic. Existing data are fragmented, largely single-marker, cross-sectional, and rarely include longitudinal sampling; no multi-hormone CCS signature has been derived and externally validated.	Research only. No hormonal panel is currently recommended for CCS phenotyping, and none has an established diagnostic cutoff.
**Mechanistic**	Studies linking hormonal profiles to endothelial function, coronary flow reserve, microvascular resistance, plaque features (OCT/IVUS), and vasomotor testing	Connect endocrine signatures to specific biological mechanisms [14,22,23].	Mechanistic. Pathway plausibility is strong across RAAS, sympathetic, endothelin and adipokine axes, but human data coupling hormonal status to invasive coronary physiology are scarce.	Research only. Intended to generate hypotheses and candidate markers, not to inform current management.
**Interventional**	RCTs of hormone-modulating strategies stratified by hormonal phenotype (e.g., low-T3, hypogonadism, high ET-1, postmenopausal high-FSH)	Identify responders and move beyond empiric class-based prescribing [34,35,41,47].	No therapeutic evidence in CCS stratified by hormonal phenotype currently exists. The underpinning data are mechanistic and observational-prognostic; available randomized data for these agents come from non-CCS populations (hypothyroidism, symptomatic hypogonadism, microvascular angina with physiological endpoints).	Investigational phenotype markers. This is the decisive gap: until such trials are performed, no hormone-guided CCS treatment strategy can be recommended, and enrolment thresholds themselves require prospective definition.
**Pragmatic re-evaluation**	Beta-blocker indication, dose, and duration in contemporary CCS without reduced EF; deprescription trials	Address discordance between guidelines and modern evidence (REBOOT, observational data) [58,62,63].	Therapeutic evidence in CCS. Contemporary randomized data (REBOOT, REDUCE-AMI) and large observational series address an established therapy; the open questions concern patient selection, dose and duration, not hormonal measurement.	Established CCS management under active re-evaluation. The proposal refines an existing guideline-endorsed treatment rather than introducing a hormone-guided one.
**Combination strategies**	Triple neurohormonal modulation in HFmrEF/HFpEF overlap with CCS; SGLT2/ARNI integration	Test threshold effects suggested by registry data [40,55,71].	Therapeutic evidence only from non-CCS populations. Component agents have randomized outcome data in HF and CKD; combined multi-pathway neurohumoral modulation has not been tested prospectively in CCS without those indications.	Potentially actionable for comorbidity-based care where HF or CKD coexists; research only as a CCS-directed strategy in their absence.
**Data science**	Integrative models combining hormones, genomics, imaging, and clinical variables to derive hormonal risk scores for CCS	Evaluate incremental value over current CCS risk tools [9,68].	Observational-prognostic and model-derived. Any such score would require demonstration of incremental discrimination, calibration and reclassification over established CCS risk models, followed by external validation.	Research only. No hormonal risk score for CCS has been derived, validated, or shown to change management or outcomes.

## Data Availability

No new data were created or analyzed in this study. Data sharing is not applicable to this narrative review. The search strategy, eligibility criteria, and selected studies that supported the evidence synthesis are available from the corresponding author on reasonable request.

## Supplementary Materials

The following supporting information can be downloaded at: https://www.mdpi.com/article/10.3390/biomedicines14091954/s1, Table S1. Full electronic search strategy; Table S2. The 34 core therapy-focused sources retained in the structured appraisal; Table S3. Directness of the available evidence for each hormonal and therapeutic axis in chronic coronary syndrome; Table S4. Established chronic coronary syndrome phenotypes and their potential endocrine–neurohormonal modifiers.

## Abbreviations

The following abbreviations are used in this manuscript:

ACEI	angiotensin-converting-enzyme inhibitor
AKI	acute kidney injury
ANOCA	angina with non-obstructive coronary arteries
ARB	angiotensin receptor blocker
ARNI	angiotensin receptor-neprilysin inhibitor
EF	ejection fraction
ET-1	endothelin-1
FSH	follicle-stimulating hormone
GH	growth hormone
HFmrEF	heart failure with mildly reduced ejection fraction
HFpEF	heart failure with preserved ejection fraction
HFrEF	heart failure with reduced ejection fraction
HRT	hormone replacement therapy
IGF-1	insulin-like growth factor-1
INOCA	ischemia with non-obstructive coronary arteries
IVUS	intravascular ultrasound
LVEF	left ventricular ejection fraction
MASLD	metabolic dysfunction-associated steatosis liver disease
MI	myocardial infarction
MRA	mineralocorticoid receptor antagonist
NPs	natriuretic peptides
NT-proBNP	N-terminal pro-B-type natriuretic peptide
OCT	optical coherence tomography
PRISMA	Preferred Reporting Items for Systematic Reviews and Meta-Analyses
RAAS	renin–angiotensin–aldosterone system
RCT	randomized controlled trial
SHBG	sex hormone-binding globulin
T3	triiodothyronine
T4	thyroxine
T.O.S.CA.	Trial of Serial Combined Assessment
TSH	thyroid-stimulating hormone
WISE	Women’s Ischemia Syndrome Evaluation
