# Supplementary material for: Hormonal Modulation in Chronic Coronary Syndrome: From Neurohormonal Activation to Endocrine Targets
Source: Biomedicines. 2026 Aug 30;14(9):1954. doi: 10.3390/biomedicines14091954 (PMC13604452; doi:10.3390/biomedicines14091954)
Supplement: Supplementary file 1 [file biomedicines-14-01954-s001.zip › biomedicines-4492705-supplementary.pdf]

Supplementary Materials

Hormonal Modulation in Chronic Coronary Syndrome: From Neurohormonal Activation to Endocrine Targets

Alina Diduța Brie, Roxana Popescu, Cristian Mornoș, Daniel Miron Brie

Table S1. Full electronic search strategy

Table S1 reports the complete search strategy underlying the structured literature appraisal summarized in Figure 1 of the main manuscript. Section A gives the search overview (databases, interfaces, coverage, exact dates searched, and limits); Sections B–E give the verbatim, line-by-line search strings for PubMed/MEDLINE, Embase, the Cochrane Library, and ScienceDirect; Section F reconciles the retrieved records with the PRISMA-style flow diagram; Section G documents supplementary retrieval methods and the methodological caveats of a specificity-weighted search.

Section A. Search overview

**Review type**	Narrative review anchored in a prespecified, structured literature appraisal; reported in accordance with SANRA and, for the identification-to-inclusion flow, adapted from the PRISMA 2020 statement. Not registered as a systematic review; no protocol was deposited in PROSPERO.
**Databases searched**	1. PubMed/MEDLINE (U.S. National Library of Medicine, pubmed.ncbi.nlm.nih.gov)<br>2. Embase (Elsevier, embase.com interface)<br>3. Cochrane Library (Wiley) — CENTRAL and Cochrane Database of Systematic Reviews (CDSR)<br>4. ScienceDirect (Elsevier, sciencedirect.com)
**Date limits (coverage)**	From database inception to 31 May 2026, inclusive.<br>PubMed/MEDLINE: 1946–31 May 2026. Embase: 1947–31 May 2026 (including MEDLINE-unique records). Cochrane CENTRAL/CDSR: inception–31 May 2026. ScienceDirect: inception–31 May 2026.
**Exact dates searched**	Initial searches executed 18 May 2026 (all four databases).<br>Final top-up search executed 31 May 2026 (all four databases); records added by the top-up search are included in the totals below.<br>Last date searched: 31 May 2026. No further records were added after this date.

**Language restriction**	English only. Applied at the database level (PubMed: English[Filter]; Embase: [english]/lim; Cochrane and ScienceDirect: language filter set to English) and confirmed at screening. Non-English records were excluded without translation.
**Species / age limits**	Humans only. Adult populations only (PubMed: Adult: 19+ years; Embase: [adult]/lim OR [middle aged]/lim OR [aged]/lim OR [very elderly]/lim). Paediatric-only studies were excluded at screening.
**Publication types excluded at the search level**	Case reports, editorials, comments, letters, and notes. In Embase, conference abstracts, conference papers, and conference reviews were additionally excluded by item type ("/it"), consistent with the decision not to include grey literature in the core evidentiary dataset.
**Structure of the strategy**	Four concept blocks combined with the Boolean operator AND:<br>(i) Population — chronic coronary syndrome / stable coronary artery disease;<br>(ii) Exposure/intervention — neurohormonal and endocrine axes and their pharmacological modulators;<br>(iii) Outcomes — mortality, MACE, myocardial infarction, heart-failure hospitalization, cardiac function, symptoms/quality of life;<br>(iv) Study design — comparative clinical designs, evidence syntheses, and consensus documents.<br>Within each block, controlled vocabulary (MeSH / Emtree) and free-text title–abstract–keyword terms were combined with OR.
**Searches performed by**	R.P. and A.D.B. (search design and execution). Titles and abstracts were screened independently in duplicate by R.P. and A.D.B.; disagreements were resolved by discussion, with D.M.B. as third reviewer.
**Reference management / deduplication**	Records were exported in RIS/NBIB format and deduplicated in EndNote 21 (Clarivate) using a two-pass routine (automatic matching on DOI, then on author–year–title), followed by manual verification of all flagged pairs.
**Total records retrieved**	851 records before deduplication; 500 unique records after removal of 351 duplicates (see Section F and Figure 1 of the main manuscript).

Section B. PubMed/MEDLINE

Interface: pubmed.ncbi.nlm.nih.gov. Coverage: 1946 to 31 May 2026. Date searched: 18 May 2026; top-up 31 May 2026. Field tags: [Mesh] = MeSH term (exploded by default); [Majr] = MeSH Major Topic; [tiab] = title/abstract; [pt] = publication type. Truncation (*) is applied only to single words, because PubMed does not support truncation within quoted phrases; singular and plural phrase variants are therefore listed explicitly.

Set	Search string as run	Records
**Block (i) — Population: chronic coronary syndrome / stable coronary artery disease**		
**#1**	"Coronary Artery Disease"[Majr] OR "Myocardial Ischemia"[Majr] OR "Coronary Disease"[Majr] OR "Angina, Stable"[Majr] OR "Angina Pectoris"[Majr] OR "Microvascular Angina"[Majr] OR "Coronary Vasospasm"[Majr] OR "Coronary Stenosis"[Majr]	134,928
**#2**	"chronic coronary syndrome"[tiab] OR "chronic coronary syndromes"[tiab] OR "chronic coronary disease"[tiab] OR "stable coronary artery disease"[tiab] OR "stable coronary disease"[tiab] OR "stable angina"[tiab] OR "chronic stable angina"[tiab] OR "stable ischemic heart disease"[tiab] OR "stable ischaemic heart disease"[tiab] OR "chronic ischemic heart disease"[tiab] OR "chronic ischaemic heart disease"[tiab] OR INOCA[tiab] OR ANOCA[tiab] OR "non-obstructive coronary"[tiab] OR "nonobstructive coronary"[tiab] OR "coronary microvascular dysfunction"[tiab] OR "vasospastic angina"[tiab] OR "microvascular angina"[tiab]	24,117
**#3**	#1 OR #2	145,902
**Block (ii) — Exposure: neurohormonal and endocrine axes**		
**#4**	RAAS and aldosterone axis:<br>"Renin-Angiotensin System"[Mesh] OR "Angiotensin-Converting Enzyme Inhibitors"[Mesh] OR "Angiotensin Receptor Antagonists"[Mesh] OR "Mineralocorticoid Receptor Antagonists"[Mesh] OR "Aldosterone"[Mesh] OR "Renin"[Mesh] OR "Angiotensin II"[Mesh] OR "ACE inhibitor"[tiab] OR "ACE inhibitors"[tiab] OR "angiotensin converting enzyme inhibitor"[tiab] OR "angiotensin converting enzyme inhibitors"[tiab] OR "angiotensin receptor blocker"[tiab] OR "angiotensin receptor blockers"[tiab] OR sartan*[tiab] OR ramipril[tiab] OR perindopril[tiab] OR enalapril[tiab] OR lisinopril[tiab] OR trandolapril[tiab] OR valsartan[tiab] OR telmisartan[tiab] OR olmesartan[tiab] OR candesartan[tiab] OR irbesartan[tiab] OR losartan[tiab] OR spironolactone[tiab] OR eplerenone[tiab] OR finerenone[tiab] OR "mineralocorticoid receptor antagonist"[tiab] OR "mineralocorticoid receptor antagonists"[tiab] OR aldosterone[tiab]	214,776
**#5**	Sympathetic–adrenal axis:<br>"Sympathetic Nervous System"[Mesh] OR "Adrenergic beta-Antagonists"[Mesh] OR "Catecholamines"[Mesh] OR "Norepinephrine"[Mesh] OR "Epinephrine"[Mesh] OR beta-blocker*[tiab] OR "beta blocker"[tiab] OR "beta blockers"[tiab] OR "beta-adrenergic blockade"[tiab] OR bisoprolol[tiab] OR metoprolol[tiab] OR carvedilol[tiab] OR nebivolol[tiab] OR atenolol[tiab] OR "sympathetic activation"[tiab] OR "sympathetic overactivity"[tiab] OR "muscle sympathetic nerve activity"[tiab]	198,652
**#6**	Natriuretic peptides and endothelin:<br>"Natriuretic Peptides"[Mesh] OR "Natriuretic Peptide, Brain"[Mesh] OR "Atrial Natriuretic Factor"[Mesh] OR "Neprilysin"[Mesh] OR "Endothelin-1"[Mesh] OR "Endothelin Receptor Antagonists"[Mesh] OR "Endothelins"[Mesh] OR BNP[tiab] OR "NT-proBNP"[tiab] OR "natriuretic peptide"[tiab] OR "natriuretic peptides"[tiab] OR neprilysin[tiab] OR sacubitril[tiab] OR "angiotensin receptor neprilysin	71,309

Set	Search string as run	Records
	inhibitor"[tiab] OR endothelin*[tiab] OR bosentan[tiab] OR macitentan[tiab] OR zibotentan[tiab]	
#7	Sex hormones:<br>"Gonadal Steroid Hormones"[Mesh] OR "Testosterone"[Mesh] OR "Estrogens"[Mesh] OR "Estrogen Replacement Therapy"[Mesh] OR "Hormone Replacement Therapy"[Mesh] OR "Menopause"[Mesh] OR "Hypogonadism"[Mesh] OR "Androgen Antagonists"[Mesh] OR "Progesterone"[Mesh] OR testosterone[tiab] OR estrogen*[tiab] OR oestrogen*[tiab] OR "sex hormone"[tiab] OR "sex hormones"[tiab] OR "sex steroid"[tiab] OR "sex steroids"[tiab] OR "androgen deprivation"[tiab] OR "menopausal hormone therapy"[tiab] OR "hormone replacement therapy"[tiab] OR hypogonadism[tiab] OR "premature menopause"[tiab]	402,118
#8	Thyroid axis:<br>"Thyroid Hormones"[Mesh] OR "Thyrotropin"[Mesh] OR "Hypothyroidism"[Mesh] OR "Hyperthyroidism"[Mesh] OR "Euthyroid Sick Syndromes"[Mesh] OR "Thyroxine"[Mesh] OR "Triiodothyronine"[Mesh] OR "thyroid hormone"[tiab] OR "thyroid hormones"[tiab] OR levothyroxine[tiab] OR liothyronine[tiab] OR "subclinical hypothyroidism"[tiab] OR "subclinical hyperthyroidism"[tiab] OR "low T3 syndrome"[tiab] OR "low triiodothyronine"[tiab] OR TSH[tiab] OR "thyroid stimulating hormone"[tiab]	233,540
#9	Cardiometabolic hormones and adipose-derived mediators:<br>"Glucagon-Like Peptide-1 Receptor Agonists"[Mesh] OR "Sodium-Glucose Transporter 2 Inhibitors"[Mesh] OR "Incretins"[Mesh] OR "Glucagon-Like Peptide 1"[Mesh] OR "Adipokines"[Mesh] OR "Leptin"[Mesh] OR "Adiponectin"[Mesh] OR "Insulin Resistance"[Mesh] OR "Human Growth Hormone"[Mesh] OR "Insulin-Like Growth Factor I"[Mesh] OR "Hydrocortisone"[Mesh] OR "Vitamin D"[Mesh] OR semaglutide[tiab] OR liraglutide[tiab] OR dulaglutide[tiab] OR tirzepatide[tiab] OR exenatide[tiab] OR "GLP-1 receptor agonist"[tiab] OR "GLP-1 receptor agonists"[tiab] OR empagliflozin[tiab] OR dapagliflozin[tiab] OR canagliflozin[tiab] OR ertugliflozin[tiab] OR "SGLT2 inhibitor"[tiab] OR "SGLT2 inhibitors"[tiab] OR leptin[tiab] OR adiponectin[tiab] OR "insulin resistance"[tiab] OR cortisol[tiab] OR "growth hormone"[tiab] OR "insulin-like growth factor"[tiab] OR "epicardial adipose"[tiab]	187,924
#10	#4 OR #5 OR #6 OR #7 OR #8 OR #9	1,178,043
**Block (iii) — Outcomes**		
#11	"Mortality"[Mesh] OR "Cause of Death"[Mesh] OR "Myocardial Infarction"[Mesh] OR "Stroke"[Mesh] OR "Heart Failure"[Mesh] OR "Hospitalization"[Mesh] OR "Myocardial Revascularization"[Mesh] OR "Ventricular Function, Left"[Mesh] OR "Quality of Life"[Mesh] OR "Exercise Tolerance"[Mesh] OR mortality[tiab] OR death*[tiab] OR survival[tiab] OR MACE[tiab] OR "major adverse cardiovascular events"[tiab] OR "major adverse cardiac events"[tiab] OR "cardiovascular outcome"[tiab] OR "cardiovascular outcomes"[tiab] OR "myocardial infarction"[tiab] OR "heart failure hospitalization"[tiab] OR "heart failure hospitalisation"[tiab] OR "ejection fraction"[tiab] OR "angina frequency"[tiab] OR "Seattle Angina Questionnaire"[tiab] OR "quality of life"[tiab] OR "exercise capacity"[tiab] OR "coronary flow reserve"[tiab]	2,904,551
**Block (iv) — Study design**		
#12	"Randomized Controlled Trial"[pt] OR "Controlled Clinical Trial"[pt] OR "Clinical Trial, Phase III"[pt] OR "Clinical Trial, Phase IV"[pt] OR "Multicenter Study"[pt] OR "Meta-Analysis"[pt] OR "Systematic Review"[pt] OR "Practice	5,612,088

Set	Search string as run	Records
	Guideline"[pt] OR "Guideline"[pt] OR "Cohort Studies"[Mesh] OR "Case-Control Studies"[Mesh] OR "Registries"[Mesh] OR "Consensus"[Mesh] OR randomi*[tiab] OR "cohort study"[tiab] OR "cohort studies"[tiab] OR registry[tiab] OR "propensity score"[tiab] OR "systematic review"[tiab] OR meta-analys*[tiab] OR "consensus statement"[tiab] OR "consensus document"[tiab]	
**Combination and limits**		
#13	#3 AND #10	6,118
#14	#13 AND #11	1,904
#15	#14 AND #12	587
#16	#15 NOT ("Case Reports"[pt] OR "Editorial"[pt] OR "Comment"[pt] OR "Letter"[pt] OR ("Animals"[Mesh] NOT "Humans"[Mesh]))	521
#17	#16 Filters: English[Filter]; Humans[Filter]; Adult: 19+ years; Publication date 1946/01/01-2026/05/31<br><br>FINAL PubMed/MEDLINE SET	312

Section C. Embase

Interface: embase.com (Elsevier), Advanced Search. Coverage: 1947 to 31 May 2026, including MEDLINE-unique records. Date searched: 18 May 2026; top-up 31 May 2026. Syntax: 'term'/exp = Emtree term exploded; 'term'/de = Emtree term not exploded; :ti,ab,kw = title, abstract, author keywords; /it = item type; /lim = limit. Truncation is denoted by *.

Set	Search string as run	Records
**Block (i) — Population**		
#1	'coronary artery disease'/exp OR 'chronic coronary syndrome'/exp OR 'stable angina pectoris'/exp OR 'ischemic heart disease'/exp OR 'coronary artery vasospasm'/exp OR 'microvascular angina'/exp OR 'coronary artery obstruction'/exp	712,455
#2	('chronic coronary syndrome' OR 'chronic coronary syndromes' OR 'chronic coronary disease' OR 'stable coronary artery disease' OR 'stable coronary disease' OR 'stable angina' OR 'chronic stable angina' OR 'stable ischemic heart disease' OR 'stable ischaemic heart disease' OR 'chronic ischemic heart disease' OR inoca OR anoca OR 'non-obstructive coronary' OR 'nonobstructive coronary' OR 'coronary microvascular dysfunction' OR 'vasospastic angina' OR 'microvascular angina'):ti,ab,kw	31,208
#3	#1 OR #2	724,061
**Block (ii) — Exposure: neurohormonal and endocrine axes**		
#4	RAAS and aldosterone axis:<br>'renin angiotensin aldosterone system'/exp OR 'dipeptidyl carboxypeptidase inhibitor'/exp OR 'angiotensin receptor antagonist'/exp OR 'mineralocorticoid antagonist'/exp OR 'aldosterone'/exp OR 'renin'/exp OR 'angiotensin II'/exp OR	331,486

Set	Search string as run	Records
	('ACE inhibitor*' OR 'angiotensin converting enzyme inhibitor*' OR 'angiotensin receptor blocker*' OR sartan* OR ramipril OR perindopril OR enalapril OR lisinopril OR trandolapril OR valsartan OR telmisartan OR olmesartan OR candesartan OR irbesartan OR losartan OR spironolactone OR eplerenone OR finerenone OR 'mineralocorticoid receptor antagonist*'):ti,ab,kw	
#5	Sympathetic–adrenal axis: 'beta adrenergic receptor blocking agent'/exp OR 'sympathetic nervous system'/exp OR 'catecholamine'/exp OR 'noradrenalin'/exp OR ('beta blocker*' OR 'beta-blocker*' OR 'beta adrenergic blockade' OR bisoprolol OR metoprolol OR carvedilol OR nebivolol OR atenolol OR 'sympathetic activation' OR 'sympathetic overactivity'):ti,ab,kw	288,904
#6	Natriuretic peptides and endothelin: 'brain natriuretic peptide'/exp OR 'atrial natriuretic factor'/exp OR 'amino terminal pro brain natriuretic peptide'/exp OR 'neprilysin'/exp OR 'endothelin 1'/exp OR 'endothelin receptor antagonist'/exp OR (bnp OR 'nt-probnp' OR 'natriuretic peptide*' OR neprilysin OR sacubitril OR endothelin* OR bosentan OR macitentan OR zibotentan):ti,ab,kw	104,772
#7	Sex hormones: 'sex hormone'/exp OR 'testosterone'/exp OR 'estrogen'/exp OR 'hormone substitution'/exp OR 'estrogen therapy'/exp OR 'menopause'/exp OR 'hypogonadism'/exp OR 'antiandrogen'/exp OR (testosterone OR estrogen* OR oestrogen* OR 'sex hormone*' OR 'sex steroid*' OR 'androgen deprivation' OR 'menopausal hormone therapy' OR 'hormone replacement therapy' OR hypogonadism OR 'premature menopause'):ti,ab,kw	556,213
#8	Thyroid axis: 'thyroid hormone'/exp OR 'thyrotropin'/exp OR 'hypothyroidism'/exp OR 'hyperthyroidism'/exp OR 'euthyroid sick syndrome'/exp OR 'levothyroxine'/exp OR 'liothyronine'/exp OR ('thyroid hormone*' OR levothyroxine OR liothyronine OR 'subclinical hypothyroidism' OR 'subclinical hyperthyroidism' OR 'low t3 syndrome' OR tsh OR 'thyroid stimulating hormone'):ti,ab,kw	312,780
#9	Cardiometabolic hormones and adipose-derived mediators: 'glucagon like peptide 1 receptor agonist'/exp OR 'sodium glucose cotransporter 2 inhibitor'/exp OR 'incretin'/exp OR 'adipokine'/exp OR 'leptin'/exp OR 'adiponectin'/exp OR 'insulin resistance'/exp OR 'growth hormone'/exp OR 'somatomedin C'/exp OR 'hydrocortisone'/exp OR 'vitamin D'/exp OR (semaglutide OR liraglutide OR dulaglutide OR tirzepatide OR exenatide OR 'glp-1 receptor agonist*' OR empagliflozin OR dapagliflozin OR canagliflozin OR ertugliflozin OR 'sglt2 inhibitor*' OR leptin OR adiponectin OR 'insulin resistance' OR cortisol OR 'growth hormone' OR 'insulin like growth factor' OR 'epicardial adipose'):ti,ab,kw	271,459
#10	#4 OR #5 OR #6 OR #7 OR #8 OR #9	1,624,038
**Block (iii) — Outcomes**		
#11	'mortality'/exp OR 'cause of death'/exp OR 'heart infarction'/exp OR 'cerebrovascular accident'/exp OR 'heart failure'/exp OR 'hospitalization'/exp OR 'heart muscle revascularization'/exp OR 'heart left ventricle ejection fraction'/exp OR 'quality of life'/exp OR 'exercise tolerance'/exp OR (mortality OR death* OR survival OR mace OR 'major adverse cardiovascular event*' OR 'major adverse cardiac event*' OR 'cardiovascular outcome*' OR 'myocardial infarction' OR 'heart failure hospitali*' OR 'ejection fraction' OR 'angina frequency' OR	4,118,662

Set	Search string as run	Records
	'seattle angina questionnaire' OR 'quality of life' OR 'exercise capacity' OR 'coronary flow reserve'):ti,ab,kw	
**Block (iv) — Study design**		
#12	'randomized controlled trial'/exp OR 'controlled clinical trial'/exp OR 'phase 3 clinical trial'/exp OR 'phase 4 clinical trial'/exp OR 'multicenter study'/exp OR 'cohort analysis'/exp OR 'case control study'/exp OR 'register'/exp OR 'meta analysis'/exp OR 'systematic review'/exp OR 'practice guideline'/exp OR 'consensus'/exp OR (randomi* OR 'cohort stud*' OR registry OR 'propensity score' OR 'systematic review' OR 'meta-analys*' OR 'consensus statement' OR 'consensus document'):ti,ab,kw	6,884,310
**Combination and limits**		
#13	#3 AND #10	18,247
#14	#13 AND #11	5,602
#15	#14 AND #12	1,433
#16	#15 NOT ('conference abstract'/it OR 'conference paper'/it OR 'conference review'/it OR 'editorial'/it OR 'note'/it OR 'letter'/it OR 'case report'/exp)	694
#17	#16 AND [english]/lim AND [humans]/lim AND ([adult]/lim OR [middle aged]/lim OR [aged]/lim OR [very elderly]/lim) AND [1947-2026]/py, with records dated after 31 May 2026 removed manually<br><br>FINAL Embase SET	358

Section D. Cochrane Library (CENTRAL and CDSR)

Interface: Wiley Cochrane Library, Advanced Search. Coverage: inception to 31 May 2026. Date searched: 18 May 2026; top-up 31 May 2026. Syntax: MeSH descriptor searches were exploded ("explode all trees"); :ti,ab,kw = title, abstract, keywords. Wildcards (*) are supported by the Wiley interface, including within phrases.

Set	Search string as run	Records
#1	MeSH descriptor: [Coronary Artery Disease] explode all trees	9,884
#2	MeSH descriptor: [Angina, Stable] explode all trees	1,342
#3	MeSH descriptor: [Myocardial Ischemia] explode all trees	24,516
#4	("chronic coronary syndrome*" OR "stable coronary artery disease" OR "stable angina" OR "chronic stable angina" OR "stable ischemic heart disease" OR "stable ischaemic heart disease" OR INOCA OR ANOCA OR "coronary microvascular dysfunction" OR "vasospastic angina"):ti,ab,kw	7,905
#5	#1 OR #2 OR #3 OR #4	28,772

Set	Search string as run	Records
#6	MeSH descriptor: [Renin-Angiotensin System] explode all trees OR MeSH descriptor: [Adrenergic beta-Antagonists] explode all trees OR MeSH descriptor: [Mineralocorticoid Receptor Antagonists] explode all trees OR MeSH descriptor: [Natriuretic Peptides] explode all trees OR MeSH descriptor: [Endothelin-1] explode all trees OR MeSH descriptor: [Gonadal Steroid Hormones] explode all trees OR MeSH descriptor: [Thyroid Hormones] explode all trees OR MeSH descriptor: [Glucagon-Like Peptide-1 Receptor Agonists] explode all trees OR MeSH descriptor: [Sodium-Glucose Transporter 2 Inhibitors] explode all trees OR MeSH descriptor: [Adipokines] explode all trees	41,207
#7	("renin-angiotensin" OR "ACE inhibitor*" OR "angiotensin receptor blocker*" OR sartan* OR spironolactone OR eplerenone OR finerenone OR "beta-blocker*" OR bisoprolol OR metoprolol OR carvedilol OR nebivolol OR "natriuretic peptide*" OR sacubitril OR endothelin* OR testosterone OR estrogen* OR oestrogen* OR "hormone replacement therapy" OR "thyroid hormone*" OR levothyroxine OR "subclinical hypothyroidism" OR "GLP-1 receptor agonist*" OR semaglutide OR liraglutide OR tirzepatide OR "SGLT2 inhibitor*" OR empagliflozin OR dapagliflozin OR leptin OR adiponectin OR cortisol):ti,ab,kw	58,996
#8	#6 OR #7	63,540
#9	(mortality OR death* OR MACE OR "major adverse cardiovascular event*" OR "myocardial infarction" OR "heart failure" OR hospitali* OR "ejection fraction" OR "quality of life" OR "exercise capacity" OR "angina frequency" OR "coronary flow reserve"):ti,ab,kw	412,309
#10	#5 AND #8 AND #9	1,046
#11	#10, limited to Cochrane Reviews (CDSR), publication date to 31 May 2026, English language<br><br>FINAL CDSR SET	3
#12	#10, limited to Trials (CENTRAL), publication date to 31 May 2026, English language, excluding clinical-trial-registry (ICTRP) and conference-proceedings records<br><br>FINAL CENTRAL SET	118
#13	#11 OR #12 — FINAL Cochrane Library SET	121

Section E. ScienceDirect

Interface: sciencedirect.com, Advanced Search. Coverage: inception to 31 May 2026. Date searched: 18 May 2026; top-up 31 May 2026. ScienceDirect does not support wildcards/truncation, controlled vocabulary, or more than eight Boolean connectors per field, and its "Title, abstract, keywords" field does not permit nested parentheses beyond one level. The strategy was therefore decomposed into four thematically restricted searches (E1–E4) run in the "Title, abstract, keywords" field, with article type limited to Research articles and Review articles, and language set to English. ScienceDirect was used as a supplementary source for full-text journal content and not as a primary index.

Set	Search string as run	Records
E1	RAAS and sympathetic axis:<br>("chronic coronary syndrome" OR "stable angina" OR "stable coronary artery	22

Set	Search string as run	Records
	`disease") AND ("beta-blocker" OR "mineralocorticoid receptor antagonist" OR "renin-angiotensin" OR "spironolactone")`	
**E2**	`Sex and thyroid hormones: ("chronic coronary syndrome" OR "stable angina" OR "stable coronary artery disease") AND ("testosterone" OR "estrogen" OR "thyroid hormone" OR "subclinical hypothyroidism")`	16
**E3**	`Cardiometabolic hormone modulators: ("chronic coronary syndrome" OR "stable angina" OR "stable coronary artery disease") AND ("GLP-1 receptor agonist" OR "SGLT2 inhibitor" OR "semaglutide" OR "empagliflozin")`	13
**E4**	`Natriuretic peptides, endothelin and adipokines: ("chronic coronary syndrome" OR "stable angina" OR "stable coronary artery disease") AND ("natriuretic peptide" OR "endothelin-1" OR "adipokine" OR "adiponectin")`	9
—	`E1 OR E2 OR E3 OR E4 (union after removal of within-platform duplicates)` `FINAL ScienceDirect SET`	60

Section F. Reconciliation of retrieved records with Figure 1

Stage	Records (n)	Corresponding Figure 1 box
PubMed/MEDLINE (final set #17)	312	Identification
Embase (final set #17)	358	Identification
Cochrane Library — CENTRAL (118) + CDSR (3)	121	Identification
ScienceDirect (E1–E4 union)	60	Identification
**Total records identified through database searching**	**851**	Records identified (n = 851)
Duplicates removed (EndNote 21, two-pass matching with manual verification)	351	—
Unique records after deduplication	500	Records after duplicates removed (n = 500)
Records screened on title and abstract (duplicate, independent screening)	500	Records screened (n = 500)
Records excluded — did not meet eligibility criteria	466	Records excluded (n = 466)

Stage	Records (n)	Corresponding Figure 1 box
Full-text records / sources assessed for eligibility	34	Full-text assessed (n = 34)
**Studies included in the narrative synthesis**	**34**	Included (n = 34)

Principal reasons for exclusion at title/abstract screening (n = 466) were: population restricted to acute coronary syndromes without chronic-phase follow-up; non-comparative design (case reports, single-arm series, narrative commentary); preclinical, in-vitro or animal work; primary intervention not acting on a hormonal or neurohormonal axis (for example isolated revascularization, antiplatelet or lipid-lowering trials without endocrine analysis); outcomes limited to surrogate biomarkers without clinical or functional endpoints; and duplicate reports of the same trial or registry, for which the most informative and/or most recent publication was retained.

Section G. Supplementary retrieval methods and methodological notes

**Supplementary retrieval**	Backward citation tracking (reference lists of retrieved reviews, guidelines and included studies) and forward citation tracking in Google Scholar were performed for all included studies, up to 31 May 2026. Records identified by these methods were used to inform the mechanistic and background narrative but were not added to the 851-record database yield reported in Figure 1, which reflects database searching only.
**Guideline sources**	Contemporary guideline and consensus documents (for example the 2024 ESC Guidelines for the management of chronic coronary syndromes) were retrieved through the database searches and, where the full text was required, from the issuing society's website. No separate systematic search of guideline repositories was undertaken.
**Grey literature**	Conference abstracts, theses, and unpublished trial-registry entries were excluded from the core evidentiary dataset (see Embase set #16). Ongoing or emerging therapies are referenced in the narrative for context only, and such sources did not contribute to the 34 studies retained for synthesis.
**Specificity-weighted design**	Population terms were entered as MeSH Major Topic (PubMed) or exploded Emtree terms combined with title/abstract/keyword phrases, and all four concept blocks were required simultaneously. This favours precision over recall and is appropriate for a narrative synthesis intended to anchor a pathophysiology-to-therapy framework, but it does not achieve the sensitivity expected of a systematic review; relevant records not indexed with the required outcome or design terms may have been missed.

**Search peer review**	The strategy was drafted by R.P., reviewed line-by-line by A.D.B., and checked against the PRESS 2015 evidence-based checklist for electronic search strategies (Boolean logic, spelling and truncation, controlled vocabulary coverage, and limits). It was not externally peer-reviewed by an information specialist.
**Registration and protocol**	No protocol was registered. Eligibility criteria and the search concepts were specified before the searches were executed but were not deposited in a public registry.
**Reproducibility**	All search strings are reported verbatim as executed. Record counts are those returned by each interface on the final search date (31 May 2026) and may differ slightly on re-execution because of ongoing database indexing, retrospective record assignment of controlled vocabulary, and platform updates.

Abbreviations: ACE, angiotensin-converting enzyme; ANOCA, angina with non-obstructive coronary arteries; BNP, B-type natriuretic peptide; CDSR, Cochrane Database of Systematic Reviews; CENTRAL, Cochrane Central Register of Controlled Trials; GLP-1, glucagon-like peptide-1; INOCA, ischaemia with non-obstructive coronary arteries; MACE, major adverse cardiovascular events; MeSH, Medical Subject Headings; NT-proBNP, N-terminal pro-B-type natriuretic peptide; PRESS, Peer Review of Electronic Search Strategies; PRISMA, Preferred Reporting Items for Systematic Reviews and Meta-Analyses; RAAS, renin–angiotensin–aldosterone system; SANRA, Scale for the Assessment of Narrative Review Articles; SGLT2, sodium–glucose cotransporter-2; TSH, thyroid-stimulating hormone.

Supplementary Materials

Hormonal Modulation in Chronic Coronary Syndrome: From Neurohormonal Activation to Endocrine Targets

Alina Diduța Brie, Roxana Popescu, Cristian Mornoș, Daniel Miron Brie

Table S2. The 34 core therapy-focused sources retained in the structured appraisal

Table S2 lists the 34 studies retained after two-stage selection from the 500 unique records screened (Figure 1 and Supplementary Table S1). These sources formed the evidentiary core of the therapy-focused sections (Sections 4–6); additional background, mechanistic and guideline citations appear throughout the narrative and account for the larger total reference count. Entries are grouped by hormonal axis and identified by their reference number in the main manuscript. The "directness" tag in the coronary-context column indicates how closely the enrolled population matches chronic coronary syndrome: direct = CCS or stable coronary artery disease; partial = mixed or high-risk atherosclerotic populations in which CCS patients predominate but are not exclusively enrolled; indirect = heart-failure or cardiometabolic populations from which inference to CCS requires explicit extrapolation. Because a formal risk-of-bias assessment was not undertaken (see Methods), this directness tag — together with study design — is the principal means by which the strength of each contribution should be judged.

Ref.	Study (first author, year, journal)	Study design	Population (n as reported)	Coronary context [directness]	Intervention / exposure	Outcome domain	Relevance to the present review
**A. Renin–angiotensin–aldosterone system and angiotensin–neprilysin axis (n = 8)**							
**24**	Yusuf S, 2000 (HOPE) N Engl J Med	Multicentre, double-blind, placebo-controlled RCT	9,297 patients ≥55 y with vascular disease or diabetes plus one additional risk factor; no HF or low EF	Established atherosclerotic CAD without heart failure [direct]	Ramipril 10 mg/day vs placebo, median 5 years	CV death, myocardial infarction, stroke (composite); all-cause mortality	Foundational demonstration that RAAS blockade reduces hard cardiovascular events in stable vascular disease beyond blood-pressure lowering; anchors the "RAAS-dominant" hormonal phenotype in Section 4.
**25**	Fox KM, 2003 (EUROPA) Lancet	Multicentre, double-blind, placebo-controlled RCT	12,218 patients with documented stable CAD and no clinical heart failure	Stable coronary artery disease, low-risk background therapy [direct]	Perindopril 8 mg/day vs placebo, mean 4.2 years	CV death, non-fatal MI, resuscitated cardiac arrest (composite)	The most CCS-specific randomized evidence for ACE inhibition; supports RAAS modulation as disease-modifying rather than purely haemodynamic therapy in stable disease.
**26**	Maneesh S, 2021 Pharmacotherapy	Comparative retrospective cohort	Adults with established coronary artery disease receiving RAAS inhibitor therapy	Chronic coronary artery disease, real-world practice [direct]	ACE inhibitors / ARBs vs non-use	Major adverse cardiovascular events; acute kidney injury	Provides real-world effectiveness and quantifies the renal safety trade-off of RAAS blockade, informing the phenotype-guided titration discussion.
**27**	Liu W, 2024 Ren Fail	Large multicentre retrospective cohort	Patients with coronary artery disease and advanced chronic kidney disease	Chronic CAD with cardiorenal comorbidity [direct]	ACEI/ARB therapy vs no RAAS inhibition	All-cause and cardiovascular mortality	Extends RAAS-blockade benefit into the cardiorenal CCS subset, where guideline evidence is thinnest; supports the cardiorenal hormonal phenotype.
**54**	Virdis A, 2025 High Blood Press Cardiovasc Prev	Evidence-based consensus document (SIIA / SIPREC)	Adults with essential hypertension and high cardiovascular risk	High-risk hypertensive population overlapping CCS [partial]	Fixed-dose ACE inhibitor + beta-blocker combinations	Blood-pressure and cardiovascular risk reduction; adherence	Contemporary consensus rationale for dual neurohormonal blockade, used to frame combined RAAS–sympathetic modulation in the therapeutic synthesis.
**55**	Solomon SD, 2019 (PARAGON-HF) N Engl J Med	Multicentre, double-blind, active-comparator RCT	4,822 patients with heart failure and LVEF ≥45%	HFpEF, largely with coronary comorbidity [indirect]	Sacubitril/valsartan vs valsartan	HF hospitalization and CV death (composite); functional class	Defines the ceiling of natriuretic-peptide potentiation in preserved ejection fraction and the boundary of extrapolation to CCS with diastolic dysfunction.
**65**	Solomon SD, 2024 (FINEARTS-HF) N Engl J Med	Multicentre, double-blind, placebo-controlled RCT	6,001 patients with HF and LVEF ≥40%	HFmrEF/HFpEF, high prevalence of ischaemic aetiology [indirect]	Finerenone (non-steroidal MRA) vs placebo	Total worsening-HF events and CV death	Key contemporary evidence for non-steroidal mineralocorticoid receptor antagonism; underpins the proposal to test finerenone in aldosterone-driven CCS phenotypes.
**66**	Bakris GL, 2020 (FIDELIO-DKD) N Engl J Med	Multicentre, double-blind, placebo-controlled RCT	5,734 patients with chronic kidney disease and type 2 diabetes	Cardiorenal-metabolic population with high CAD prevalence [indirect]	Finerenone vs placebo on background RAAS blockade	Kidney failure progression; secondary cardiovascular composite	Establishes cardiorenal risk reduction from MRA therapy in a phenotype that overlaps substantially with cardiorenal CCS.

Ref.	Study (first author, year, journal)	Study design	Population (n as reported)	Coronary context [directness]	Intervention / exposure	Outcome domain	Relevance to the present review
**B. Sympathetic–adrenal axis and beta-adrenergic blockade (n = 6)**							
**59**	Yndigegn T, 2024 (REDUCE-AMI) N Engl J Med	Registry-based, open-label, randomized clinical trial	5,020 patients after acute MI with LVEF ≥50%	Post-MI transition to the chronic phase [partial]	Long-term beta-blocker therapy vs no beta-blocker	All-cause death or new MI (composite)	Central evidence that routine beta-blockade confers no outcome benefit when ejection fraction is preserved; the empirical basis for phenotype-restricted rather than universal sympathetic blockade.
**58**	Rossello X, 2026 (REBOOT) Eur Heart J Cardiovasc Pharmacother	Prespecified landmark analysis of a randomized trial	Patients with acute and chronic coronary syndromes without reduced ejection fraction	Acute-to-chronic coronary syndrome continuum [partial]	Beta-blocker continuation vs discontinuation	Death, reinfarction, HF hospitalization over landmark intervals	Most recent randomized signal on time-dependent beta-blocker benefit; directly informs the "when to stop" question in stable CCS management.
**61**	Godoy LC, 2023 J Am Coll Cardiol	Population-based observational cohort with propensity adjustment	Patients with stable ischaemic heart disease undergoing coronary angiography	Stable ischaemic heart disease [direct]	Beta-blocker therapy vs none, stratified by prior MI and LV function	All-cause mortality; cardiovascular hospitalization	Large real-world dataset showing benefit concentrated in the prior-MI / reduced-EF subgroup, supporting phenotype stratification of sympathetic modulation.
**63**	Park J, 2022 Korean Circ J	Retrospective observational cohort	Patients with chronic coronary artery disease treated with PCI	Chronic CAD after revascularization [direct]	Beta-blocker therapy after PCI vs no beta-blocker	Composite of death, MI and repeat revascularization	Addresses the contemporary post-PCI CCS population in which the historical beta-blocker evidence base is least applicable.
**60**	Oba Y, 2023 Circ J	Real-world multimodal database cohort	Patients with coronary artery disease under continuous heart-rate monitoring	Chronic CAD in routine care [direct]	Beta-blocker dose and achieved heart rate	Composite cardiovascular events; prognosis by heart rate	Links sympathetic tone (achieved heart rate) rather than drug exposure alone to outcome, supporting heart rate as a candidate phenotyping variable.
**62**	Liang J, 2025 Rev Cardiovasc Med	Systematic review and meta-analysis of observational studies	Pooled cohorts of patients with stable coronary artery disease	Stable coronary artery disease [direct]	Beta-blocker therapy vs no beta-blocker therapy	All-cause and cardiovascular mortality; MACE	Quantitative synthesis confirming heterogeneous, subgroup-dependent benefit; used to justify the shift from routine to phenotype-guided beta-blockade.
**C. Natriuretic peptide and endothelin axes (n = 3)**							
**32**	Omland T, 2007 (PEACE) J Am Coll Cardiol	Prospective biomarker substudy of a randomized trial	Approximately 3,700 patients with stable CAD and preserved LV function	Stable coronary artery disease [direct]	B-type natriuretic peptide as a prognostic exposure	Cardiovascular death, MI, stroke, heart failure	Demonstrates that counter-regulatory natriuretic peptide activation carries prognostic information in CCS independent of LV function; supports NP-based phenotyping.
**33**	Bibbins-Domingo K, 2007 JAMA	Prospective observational cohort (Heart and Soul Study)	987 outpatients with stable coronary heart disease	Stable coronary heart disease [direct]	NT-proBNP concentration as a prognostic exposure	Cardiovascular events and all-cause mortality	Independent confirmation across a second CCS cohort that natriuretic peptide elevation identifies residual risk not captured by anatomy or ejection fraction.
**35**	Morrow AJ, 2020 (PRIZE) Am Heart J	Rationale and design of a randomized, placebo-controlled trial	Adults with microvascular angina and objective evidence of coronary microvascular dysfunction	INOCA / microvascular angina [direct]	Zibotentan (endothelin-A receptor antagonist) vs placebo	Treadmill exercise duration; angina and quality-of-life measures	The only randomized endothelin-targeted programme in the CCS/INOCA phenotype; the exemplar for the phenotype-stratified trial design proposed in the research agenda.
**D. Sex hormones (n = 6)**							

Ref.	Study (first author, year, journal)	Study design	Population (n as reported)	Coronary context [directness]	Intervention / exposure	Outcome domain	Relevance to the present review
**37**	Manson JE, 2017 (WHI) JAMA	Long-term follow-up of two randomized placebo-controlled trials	27,347 postmenopausal women, 18 years of cumulative follow-up	Primary-prevention population, subsequent coronary events [partial]	Conjugated equine oestrogens ± medroxyprogesterone vs placebo	All-cause and cause-specific mortality, including cardiovascular	Definitive randomized evidence that timing and formulation, not oestrogen exposure per se, determine cardiovascular effect; grounds the timing-hypothesis discussion.
**67**	Lincoff AM, 2023 (TRAVERSE) N Engl J Med	Multicentre, double-blind, placebo-controlled non-inferiority RCT	5,246 men aged 45–80 y with hypogonadism and established CVD or high CV risk	Established or high-risk atherosclerotic disease [direct]	Transdermal testosterone vs placebo, mean 33 months	MACE (CV death, non-fatal MI, non-fatal stroke)	Resolves the long-standing safety question for androgen replacement in men with coronary disease and permits testosterone deficiency to be treated as a modifiable CCS phenotype rather than a contraindication.
**38**	Zhao D, 2018 J Am Coll Cardiol	Prospective multi-ethnic observational cohort (MESA)	Postmenopausal women free of cardiovascular disease at baseline	Incident coronary heart disease [partial]	Endogenous testosterone, oestradiol and their ratio	Incident CHD, heart failure and total cardiovascular disease	Shows that a more androgenic endogenous profile predicts incident coronary events in women, providing the mechanistic bridge for sex-specific hormonal phenotyping.
**39**	Yeap BB, 2021 J Clin Endocrinol Metab	Large prospective population cohort	Community-dwelling middle-aged and older men	General male population with incident coronary events [partial]	Serum testosterone and sex hormone-binding globulin	All-cause and cardiovascular mortality	Establishes the inverse testosterone–mortality gradient at population scale, defining the exposure–risk curve against which TRAVERSE-era replacement decisions are made.
**43**	Pugh PJ, 2004 Heart	Randomized, double-blind, placebo-controlled pilot trial	Men with chronic heart failure (small mechanistic sample)	Chronic heart failure, predominantly ischaemic [indirect]	Transdermal testosterone vs placebo	Exercise capacity (shuttle walk test); symptom class	Earliest randomized functional signal for androgen therapy in cardiac disease; cited as proof-of-concept and as an illustration of the small-sample limitation in this axis.
**45**	Glisic M, 2018 Maturitas	Systematic review and meta-analysis of randomized controlled trials	Postmenopausal women enrolled in randomized supplementation trials	Cardiometabolic risk substrate relevant to CCS [indirect]	Phytoestrogen supplementation vs control	Body composition and adiposity measures	Included as the quantitative evidence base for non-pharmacological oestrogen-receptor modulation and its effect on the adiposity substrate linking sex hormones to CCS risk.
**E. Thyroid axis (n = 4)**							
**46**	Razvi S, 2018 J Am Coll Cardiol	State-of-the-art evidence synthesis	Adults with overt and subclinical thyroid dysfunction	Thyroid dysfunction across the cardiovascular spectrum [partial]	Thyroid hormone status and levothyroxine replacement	Cardiovascular morbidity, mortality and cardiac function	Primary reference for the thyroid–cardiovascular interface; supplies the effect-direction framework used to define the hypothyroid CCS phenotype.
**47**	Iervasi G, 2003 Circulation	Prospective observational cohort	573 patients hospitalized with cardiac disease	Ischaemic and non-ischaemic heart disease [partial]	Low-T3 syndrome (non-thyroidal illness pattern)	Cardiac and all-cause mortality	The reference study establishing low free T3 as a strong independent mortality predictor in cardiac disease; the empirical core of the low-T3 phenotype.
**49**	Li F, 2022 (RCSCD-TCM) Endocrine	Large hospital-based observational cohort	Patients with coronary heart disease in a Chinese multicentre registry	Coronary heart disease [direct]	Central and peripheral thyroid hormone sensitivity indices	Incident arrhythmia (including atrial fibrillation)	Moves the thyroid axis beyond simple TSH categories to sensitivity indices, supporting a graded rather than dichotomous hormonal phenotype in CCS.
**30**	Wang L, 2024 Chin Gen Pract	Single-centre retrospective cohort	Patients with coronary artery disease after percutaneous coronary intervention	Chronic CAD after revascularization [direct]	Thyroid dysfunction (overt and subclinical)	Cardiac structure and left ventricular function on echocardiography	Provides directly CCS-relevant evidence that thyroid status modifies post-PCI remodelling, linking the thyroid axis to a functional rather than purely biochemical endpoint.

Ref.	Study (first author, year, journal)	Study design	Population (n as reported)	Coronary context [directness]	Intervention / exposure	Outcome domain	Relevance to the present review
**F. Cardiometabolic hormones: incretin and SGLT2 pathways (n = 4)**							
**68**	Marso SP, 2016 (LEADER) N Engl J Med	Multicentre, double-blind, placebo-controlled cardiovascular outcome RCT	9,340 patients with type 2 diabetes at high cardiovascular risk	Predominantly established atherosclerotic disease [partial]	Liraglutide (GLP-1 receptor agonist) vs placebo	MACE; cardiovascular and all-cause mortality	Establishes incretin-axis modulation as outcome-modifying in patients whose profile overlaps the cardiometabolic CCS phenotype.
**69**	Zinman B, 2015 (EMPA-REG OUTCOME) N Engl J Med	Multicentre, double-blind, placebo-controlled cardiovascular outcome RCT	7,020 patients with type 2 diabetes and established cardiovascular disease	Established atherosclerotic cardiovascular disease [partial]	Empagliflozin vs placebo	MACE; cardiovascular death; HF hospitalization	The index trial for SGLT2 inhibition; its early mortality separation motivates the neurohormonal rather than glycaemic interpretation adopted in this review.
**70**	Packer M, 2020 (EMPEROR-Reduced) N Engl J Med	Multicentre, double-blind, placebo-controlled RCT	3,730 patients with heart failure and reduced ejection fraction	HFrEF, majority of ischaemic aetiology [indirect]	Empagliflozin vs placebo on background neurohormonal therapy	CV death or HF hospitalization; renal composite	Demonstrates additive benefit on top of full RAAS and beta-blocker therapy, supporting the multi-axis rather than single-axis model of hormonal modulation.
**71**	Cherney DZI, 2025 (VERTIS CV) Kidney Dis (Basel)	Prespecified subgroup analysis of a randomized cardiovascular outcome trial	8,246 patients with type 2 diabetes and atherosclerotic cardiovascular disease	Established atherosclerotic cardiovascular disease [partial]	Ertugliflozin vs placebo, stratified by baseline RAAS inhibitor / MRA use	Cardiovascular and renal composite outcomes	The only included analysis that formally tests interaction between two hormonal axes, and therefore directly supports the combinatorial phenotype concept.
**G. Integrated multi-axis modulation and guideline framework (n = 3)**							
**72**	Cittadini A, 2021 (T.O.S.CA. Registry) Eur J Prev Cardiol	Prospective multicentre observational registry	Patients with chronic heart failure systematically screened for hormonal deficiency	Chronic heart failure, largely ischaemic [indirect]	Multiple hormonal and metabolic deficiency syndrome (composite endocrine exposure)	All-cause mortality and cardiovascular hospitalization	The closest existing empirical analogue to the hormonal-phenotype framework proposed here: demonstrates that the number of concurrent deficiencies, not any single hormone, predicts outcome.
**53**	Stewart MH, 2023 Cardiovasc Drugs Ther	Structured evidence synthesis	Adults across the cardiovascular disease spectrum	Coronary disease, hypertension and heart failure [partial]	Combined beta-blocker and ACE inhibitor therapy	Mortality, cardiovascular events and haemodynamic endpoints	Summarizes the evidence for dual sympathetic–RAAS blockade and its diminishing returns, informing the discussion of how many axes should be targeted simultaneously.
**1**	Vrints C, 2024 Eur Heart J	Evidence-based clinical practice guideline (ESC)	Adults with chronic coronary syndromes	Full CCS spectrum, including INOCA/ANOCA and vasospastic angina [direct]	Guideline-endorsed neurohormonal and cardiometabolic therapy	Class and level of recommendation for mortality, MACE and symptom endpoints	The comparator framework for the review: defines current anatomy- and comorbidity-based classification, against which the proposed hormonal-phenotype approach is positioned as complementary.

Distribution of the 34 included sources by study design

Study design category	n	Reference numbers in the main manuscript
Randomized controlled trials and prespecified analyses of randomized trials	15	Refs 24, 25, 32, 37, 43, 55, 58, 59, 65, 66, 67, 68, 69, 70, 71

Study design category	n	Reference numbers in the main manuscript
Randomized trial rationale-and-design publication	1	Ref 35 (PRIZE)
Prospective and retrospective observational cohorts and registries	12	Refs 26, 27, 30, 33, 38, 39, 47, 49, 60, 61, 63, 72
Systematic reviews and meta-analyses	2	Refs 45, 62
Structured evidence syntheses / state-of-the-art reviews	2	Refs 46, 53
Guidelines and evidence-based consensus documents	2	Refs 1, 54
**Total**	**34**	—

Distribution of the 34 included sources by hormonal axis and directness to CCS

Hormonal axis	Sources (n)	Direct to CCS	Partial	Indirect	Comment on the evidence base
A. RAAS, aldosterone and angiotensin–neprilysin axis	8	4	1	3	Two landmark CCS-specific RCTs (HOPE, EUROPA) with contemporary MRA evidence extrapolated from HF and cardiorenal trials.
B. Sympathetic–adrenal axis and beta-blockade	6	4	2	0	The most CCS-direct evidence base in the review, and the axis where randomized data most clearly support restricting rather than broadening therapy.
C. Natriuretic peptide and endothelin axes	3	3	0	0	Prognostic evidence is strong and CCS-specific; interventional evidence is limited to one ongoing randomized programme.
D. Sex hormones	6	1	3	2	One large randomized safety trial in men; evidence in women remains observational or derived from primary-prevention trials.
E. Thyroid axis	4	2	2	0	Entirely observational; no randomized trial of thyroid hormone modulation with cardiovascular endpoints in CCS was identified.
F. Incretin and SGLT2 pathways	4	0	3	1	Uniformly randomized and high-quality, but no trial enrolled a CCS population defined as such — the largest directness gap in the review.
G. Integrated multi-axis modulation and guidelines	3	1	1	1	Includes the only registry that treats concurrent hormonal deficiencies as a composite exposure (T.O.S.CA.).
**Total**	**34**	**15**	**12**	**7**	—

Notes. Reference numbers correspond to the numbered reference list of the main manuscript. Sample sizes are those reported in the primary publication; where a source is a substudy, landmark or subgroup analysis, the population of the parent trial is given. Studies are assigned to a single hormonal axis on the basis of their primary intervention or exposure, although several act on more than one axis (for example, sacubitril/valsartan on both the RAAS and natriuretic peptide systems, and SGLT2 inhibitors on sympathetic, natriuretic and metabolic signalling); such overlap is discussed in the corresponding sections of the main text. No formal risk-of-bias grading or certainty-of-evidence assessment was performed, consistent with the narrative design of the review.

Abbreviations: ACE, angiotensin-converting enzyme; ACEI, angiotensin-converting enzyme inhibitor; ANOCA, angina with non-obstructive coronary arteries; ARB, angiotensin receptor blocker; CAD, coronary artery disease; CCS, chronic coronary syndrome; CHD, coronary heart disease; CV, cardiovascular; EF, ejection fraction; GLP-1, glucagon-like peptide-1; HF, heart failure; HFmrEF, heart failure with mildly reduced ejection fraction; HFpEF,

heart failure with preserved ejection fraction; HFrEF, heart failure with reduced ejection fraction; INOCA, ischaemia with non-obstructive coronary arteries; LV, left ventricular; LVEF, left ventricular ejection fraction; MACE, major adverse cardiovascular events; MI, myocardial infarction; MRA, mineralocorticoid receptor antagonist; NT-proBNP, N-terminal pro-B-type natriuretic peptide; PCI, percutaneous coronary intervention; RAAS, renin–angiotensin–aldosterone system; RCT, randomized controlled trial; SGLT2, sodium–glucose cotransporter-2; T3, triiodothyronine; TSH, thyroid-stimulating hormone.

Supplementary Materials

Hormonal Modulation in Chronic Coronary Syndrome: From Neurohormonal Activation to Endocrine Targets

Alina Diduța Brie, Roxana Popescu, Cristian Mornoș, Daniel Miron Brie

Table S3. Directness of the available evidence for each hormonal and therapeutic axis in chronic coronary syndrome

Table S3 maps, for each hormonal or therapeutic axis discussed in the review, the extent to which the supporting evidence was generated in a chronic coronary syndrome population rather than in an adjacent population from which inference must be extrapolated. Four evidence contexts are distinguished: direct CCS cohorts; stable or chronic coronary artery disease populations; post-myocardial infarction or post-acute coronary syndrome populations; and heart failure cohorts in which coronary disease is prevalent. The final column states the practical interpretation for CCS management. The table makes explicit where the hormonal-phenotype framework rests on direct evidence and where it remains hypothesis-generating; it is not a formal certainty-of-evidence assessment, which was not undertaken (see Methods).

Hormonal / therapeutic axis	Direct CCS evidence	Stable CAD / chronic CAD evidence	Post-MI / post-ACS evidence	HF–CAD overlap evidence	Interpretation for CCS
**RAAS inhibition**	Limited CCS-specific evidence; relevant to selected post-PCI, CKD, hypertensive, and diffuse CAD phenotypes	Stronger historical evidence from stable CAD and high-risk vascular populations, including HOPE and EUROPA	Supports post-MI remodeling and secondary prevention in appropriate subgroups	Strong evidence when heart failure or LV dysfunction is present	Use according to established CCS comorbidities and conventional indications; a RAAS-dominant CCS phenotype remains conceptual
**Beta-blockers**	Limited direct evidence in CCS, with potential symptom benefit in angina and selected microvascular phenotypes	Heterogeneous observational evidence in stable ischemic heart disease	Contemporary post-MI trials with preserved EF question routine long-term prognostic use	Established in HFrEF, but this indication cannot be generalized to CCS without heart failure	Individualize for angina, heart-rate control, arrhythmia, recent MI, or reduced EF; avoid assuming a universal prognostic benefit in stable CCS
**MRAs and ARNI**	No convincing CCS-specific outcome evidence	Limited surrogate or high-risk CAD data	Limited post-MI applicability outside LV dysfunction or heart failure	Strongest evidence in HFrEF; selected evidence in HFmrEF/HFpEF and CKD–diabetes populations	Consider only when a concomitant heart failure, CKD, diabetic kidney disease, or other guideline-supported indication is present; not established as CCS-directed treatment
**Natriuretic peptides**	May have prognostic relevance in stable coronary presentations	Prognostic data in stable CAD, including PEACE and Heart and Soul	Prognostic role after MI, especially with LV dysfunction or remodeling	Strong diagnostic and prognostic relevance in heart failure	Useful as markers of myocardial stress or occult heart failure; not validated as biomarkers guiding hormonal therapy in CCS
**Endothelin pathway**	Most relevant to CMD, ANOCA and INOCA and to vasomotor phenotypes; therapeutic evidence remains investigational	Mainly mechanistic and observational evidence	Little direct relevance except in selected vascular or remodeling contexts	Heart failure data do not establish a role in CCS	An attractive mechanistic target for microvascular and vasomotor CCS, but not a routine treatment strategy
**Sex-hormone interventions**	Sparse direct CCS evidence	Mainly observational CAD data and selected hormonal studies	Limited and indirect post-MI evidence	Heart failure data are not directly applicable	Treat endocrine disorders for standard clinical indications; do not present hormone therapy as a CCS intervention
**Thyroid interventions**	Limited observational data in CCS and post-PCI settings	Observational CAD data, including low-T3 and subclinical thyroid dysfunction	Prognostic post-MI data; interventional evidence is limited	Frequent in heart failure, but correction has not been established as a CCS treatment	Assess and treat thyroid disease according to endocrine indications; cardiovascular outcome benefit in CCS remains unproven
**GLP-1 receptor agonists and SGLT2 inhibitors**	Direct CCS-specific trial evidence is limited	Cardiovascular-outcome trials include many patients with	Some populations include prior MI, but results are not specific to post-MI CCS	Particularly robust SGLT2-inhibitor evidence in heart failure and GLP-1 receptor	Use according to diabetes, obesity, CKD, and heart failure indications; their role in

Hormonal / therapeutic axis	Direct CCS evidence	Stable CAD / chronic CAD evidence	Post-MI / post-ACS evidence	HF–CAD overlap evidence	Interpretation for CCS
		established ASCVD or stable CAD		agonist evidence in diabetes and obesity with ASCVD	phenotype-matched CCS treatment remains to be tested

Notes. "Direct CCS evidence" denotes data generated in populations enrolled and characterized as chronic coronary syndrome or stable coronary artery disease without heart failure; the remaining columns denote adjacent populations requiring explicit extrapolation. Several agents act on more than one axis — for example sacubitril/valsartan on both the renin–angiotensin–aldosterone and natriuretic peptide systems, and SGLT2 inhibitors on sympathetic, natriuretic and metabolic signalling — and are listed under their principal mechanism of action. The interpretations in the final column reflect the current evidence base and should be read alongside the prevailing guideline recommendations rather than as independent practice guidance.

Abbreviations: ACS, acute coronary syndrome; ANOCA, angina with non-obstructive coronary arteries; ARNI, angiotensin receptor–neprilysin inhibitor; ASCVD, atherosclerotic cardiovascular disease; CAD, coronary artery disease; CCS, chronic coronary syndrome; CKD, chronic kidney disease; CMD, coronary microvascular dysfunction; EF, ejection fraction; EUROPA, European Trial on Reduction of Cardiac Events with Perindopril in Stable Coronary Artery Disease; GLP-1, glucagon-like peptide-1; HF, heart failure; HFmrEF, heart failure with mildly reduced ejection fraction; HFpEF, heart failure with preserved ejection fraction; HFrEF, heart failure with reduced ejection fraction; HOPE, Heart Outcomes Prevention Evaluation; INOCA, ischemia with non-obstructive coronary arteries; LV, left ventricular; MI, myocardial infarction; MRA, mineralocorticoid receptor antagonist; PCI, percutaneous coronary intervention; PEACE, Prevention of Events with Angiotensin Converting Enzyme Inhibition; RAAS, renin–angiotensin–aldosterone system; SGLT2, sodium–glucose cotransporter-2; T3, triiodothyronine.

Supplementary Materials

Hormonal Modulation in Chronic Coronary Syndrome: From Neurohormonal Activation to Endocrine Targets

Alina Diduța Brie, Roxana Popescu, Cristian Mornoș, Daniel Miron Brie

Table S4. Established chronic coronary syndrome phenotypes and their potential endocrine–neurohormonal modifiers

Table S4 sets the hormonal-phenotype framework proposed in this review against the phenotypes already established in clinical practice and guideline documents. For each recognized CCS phenotype it lists the dominant clinical and coronary features, the endocrine and neurohormonal pathways that may modify it, the assessments relevant in routine care, and the evidence status of any hormone-directed implication. The table shows where hormonal profiling might add information to existing phenotyping, not a parallel classification or a modification of guideline-directed management.

Established CCS phenotype	Dominant clinical and coronary features	Potential endocrine–neurohormonal modifiers	Relevant assessments	Implications and evidence status
**Obstructive focal CAD**	Flow-limiting epicardial stenosis, exertional angina, demonstrable ischemia	RAAS activation, sympathetic drive, cardiometabolic dysfunction, inflammation	Blood pressure, heart rate, diabetes and obesity status, renal function, lipid profile	Guideline-directed antianginal, preventive and revascularization strategies remain primary; hormonal profiling is adjunctive and unvalidated
**Diffuse atherosclerotic CAD**	Diffuse plaque burden, residual ischemia, recurrent symptoms or events despite revascularization	RAAS and aldosterone activation, adipokine imbalance, insulin resistance, inflammation	Blood pressure, CKD, diabetes, obesity, natriuretic peptides when ventricular stress is suspected	Supports aggressive risk-factor modification; no phenotype-targeted endocrine strategy has been validated
**Post-MI CCS**	Prior infarction, scar and remodeling risk, variable LV dysfunction, recurrent angina or heart failure symptoms	RAAS and sympathetic activation, natriuretic peptide elevation, thyroid dysfunction, cardiometabolic risk	LVEF, NT-proBNP, rhythm assessment, renal function, thyroid testing when clinically indicated	Apply secondary prevention and post-MI or heart failure indications according to LVEF and clinical status; do not generalize heart failure evidence to all post-MI CCS
**Post-PCI or post-CABG CCS**	Recurrent angina, progression of native disease, graft disease, in-stent restenosis, functional ischemia	RAAS activation, inflammation, autonomic dysfunction, cardiometabolic risk	Ischemia testing, coronary imaging and physiology as appropriate, renal and metabolic evaluation	Identify structural versus functional causes of symptoms first; hormonal mechanisms are potential contributors, not primary diagnostic labels
**ANOCA and INOCA**	Angina or ischemia without obstructive epicardial disease	Endothelial dysfunction, sympathetic activation, sex-hormone influences, adipokine and inflammatory dysregulation	Coronary functional testing, endothelial and vasomotor assessment, metabolic risk profile	Endotype-guided antianginal treatment remains central; endocrine targets remain mechanistic or investigational
**Coronary microvascular dysfunction**	Reduced coronary flow reserve or increased microvascular resistance, often with exertional symptoms	Endothelin excess, RAAS activation, autonomic imbalance, insulin resistance, obesity-related inflammation	Invasive or non-invasive microvascular testing, blood pressure, glycemic and metabolic evaluation	Strong rationale for integrating metabolic and vascular risk; endothelin-directed therapy and hormone-guided management are not established
**Vasospastic angina**	Epicardial or microvascular spasm, often with rest symptoms and dynamic ECG changes	Autonomic imbalance, endothelial dysfunction, possible sex-hormonal and inflammatory influences	Provocation testing where indicated, medication and trigger review	Calcium-channel blocker and nitrate-based treatment is primary; neurohormonal approaches remain adjunctive or speculative
**CCS with HFpEF overlap**	Dyspnea or angina with diastolic dysfunction, obesity, hypertension, diabetes, CKD, elevated filling pressures	Natriuretic peptide dysregulation, RAAS and aldosterone activation, adipose dysfunction, insulin resistance, inflammation	Echocardiography, natriuretic peptides, renal function, metabolic evaluation	Treat confirmed HFpEF and comorbid conditions according to heart failure guidelines; benefits cannot be attributed to CCS alone
**Cardiometabolic CCS**	Diabetes, obesity, MASLD, CKD, diffuse or microvascular disease, high residual risk	Insulin resistance, incretin dysregulation, leptin and adiponectin	Glycemia, body composition, renal function, hepatic and metabolic evaluation	GLP-1 receptor agonists and SGLT2 inhibitors should be used according to established diabetes, obesity, CKD and heart failure indications; phenotype-matched use in CCS needs testing

Established CCS phenotype	Dominant clinical and coronary features	Potential endocrine–neurohormonal modifiers	Relevant assessments	Implications and evidence status
		imbalance, RAAS activation, inflammation		

Notes. The endocrine–neurohormonal modifiers listed are candidate contributors identified from mechanistic and observational data; none has been validated as a treatment-selection criterion. Phenotypes are those recognized in the 2024 ESC Guidelines for the management of chronic coronary syndromes and in related consensus documents; they are not mutually exclusive, and individual patients frequently satisfy more than one category (for example post-MI CCS with HFpEF overlap, or diffuse atherosclerotic CAD in a cardiometabolic phenotype). The assessments listed are those already indicated in routine CCS care; no additional hormonal testing is proposed as a standard of care. Statements in the final column reflect the current evidence base and should be read alongside prevailing guideline recommendations rather than as independent practice guidance.

Abbreviations: ANOCA, angina with non-obstructive coronary arteries; CABG, coronary artery bypass grafting; CAD, coronary artery disease; CCS, chronic coronary syndrome; ECG, electrocardiogram; GLP-1, glucagon-like peptide-1; HFpEF, heart failure with preserved ejection fraction; INOCA, ischemia with non-obstructive coronary arteries; LV, left ventricular; LVEF, left ventricular ejection fraction; MASLD, metabolic dysfunction-associated steatotic liver disease; MI, myocardial infarction; NT-proBNP, N-terminal pro-B-type natriuretic peptide; PCI, percutaneous coronary intervention; RAAS, renin–angiotensin–aldosterone system; SGLT2, sodium–glucose cotransporter-2.
